# Myeloid-like B cells boost emergency myelopoiesis through IL-10 production during infection

**DOI:** 10.1084/jem.20221221

**Published:** 2023-01-31

**Authors:** Masashi Kanayama, Yuta Izumi, Megumi Akiyama, Toyoki Hayashi, Koji Atarashi, Axel Roers, Taku Sato, Toshiaki Ohteki

**Affiliations:** 1 Department of Biodefense Research, Medical Research Institute, Tokyo Medical and Dental University, Tokyo, Japan; 2 Department of Microbiology and Immunology, Keio University School of Medicine, Tokyo, Japan; 3 Institute for Immunology, Heidelberg University Hospital, Heidelberg, Germany

## Abstract

Emergency myelopoiesis (EM) is a hematopoietic response against systemic infections that quickly supplies innate immune cells. As lymphopoiesis is strongly suppressed during EM, the role of lymphocytes in that process has not received much attention. Here, we found that myeloid-like B cells (M-B cells), which express myeloid markers, emerge in the bone marrow (BM) after the induction of EM. M-B cells were mainly derived from pre-B cells and preferentially expressed IL-10, which directly stimulates hematopoietic progenitors to enhance their survival and myeloid-biased differentiation. Indeed, lacking IL-10 in B cells, blocking IL-10 in the BM with a neutralizing antibody, and deleting the IL-10 receptor in hematopoietic progenitors significantly suppressed EM, which failed to clear microbes in a cecal ligation and puncture model. Thus, a distinct B cell subset generated during infection plays a pivotal role in boosting EM, which suggests the on-demand reinforcement of EM by adaptive immune cells.

## Teaser

Collaboration between myeloid and lymphoid lineages on hematopoiesis has not been fully elucidated. Here, we showed that during systemic infection, a unique B cell subset expressing myeloid markers emerged to enhance on-demand emergency myelopoiesis by producing IL-10.

## Graphical Abstract

**Figure:**
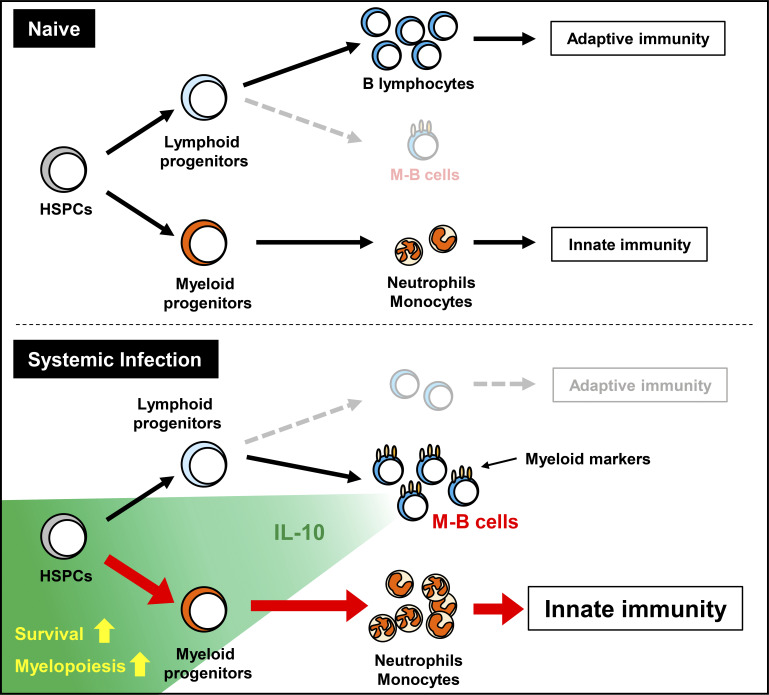

## Introduction

The hematopoietic system constantly supplies both myeloid and lymphoid cells under steady-state conditions. However, upon infection, hematopoiesis is biased toward myelopoiesis to quickly counter invading pathogens, a process known as “emergency myelopoiesis” (EM; Chavakis et al., 2019; Manz and Boettcher, 2014). Although innate immune cells play crucial roles early in EM by phagocytosing the pathogens, releasing pro-inflammatory cytokines, and triggering adaptive immunity, the mechanism(s) regulating EM are not fully understood. Pattern-recognition receptors sense pathogen-associated molecular patterns and induce the production of proinflammatory cytokines, including TNF-α, IL-6, and IL-1β. Although these inflammatory factors induce EM (Boettcher et al., 2012; Chavakis et al., 2019; Chiba et al., 2018), they simultaneously cause cell death and tissue damage (Allan et al., 2005; Bedoui et al., 2020; Hagar et al., 2013; Kang and Kishimoto, 2021; Karki et al., 2021; Tang et al., 2019). Under such a complex environment, it is largely unknown how the hematopoietic system maintains its functional integrity to strategically respond to infections.

IL-10 is a representative anti-inflammatory cytokine, which also suppresses cell death and tissue injury (Couper et al., 2008). In addition, IL-10 plays a role in maintaining the self-renewal of hematopoietic stem cells during physiological hematopoiesis (Camacho et al., 2020). Bone marrow (BM)–resident regulatory T cells (Tregs) and plasma cells have been reported as IL-10 producers in naive and in BM-transplanted mice (Fujisaki et al., 2011; Meng et al., 2019). Regulatory B cells are characterized by the capacity to produce anti-inflammatory cytokines, such as IL-10, and coordinate the pathogenesis of multiple inflammatory disorders (Mauri, 2021; Ran et al., 2020). In addition, in infection-free mice, genetic overexpression of IL-10 in stromal cells indirectly stimulates T cells to produce IFN-γ, which acts on hematopoietic progenitors to induce myelopoiesis (Cardoso et al., 2021). Although these cells are possible IL-10 producers, the importance of IL-10 in the BM during infection-induced EM remains unknown. In this context, the roles of IL-10 in the host defense against infections have been reported. IL-10–mediated signaling prevents antimicrobial immunity and the clearance of pathogens (Bermudez and Champsi, 1993; Brooks et al., 2006; Ejrnaes et al., 2006; Kessler et al., 2017; Murray and Young, 1999). IL-10 protects the host from infection-induced excessive inflammation and tissue injury (Gaddi et al., 2012; Gazzinelli et al., 1996; Li et al., 1999). However, as the number of lymphocytes in the BM is dramatically decreased during EM due to the suppression of lymphopoiesis and their mobilization to the periphery (Cain et al., 2009; Glatman Zaretsky et al., 2014; Kanayama et al., 2020), the role of lymphocytes in the induction of EM has never been noticed or tested.

In this study, we found the appearance of unique B cells that express myeloid markers, such as CD11b, F4/80, and Ly6C, and myeloid cell–related genes in the BM of mice early after LPS injection (termed myeloid-like B cells [M-B cells]). M-B cells are phenotypically distinct from other CD11b^+^ B cell subsets such as B1 cells, age-associated B cells (ABCs), and CD11b^+^ pro-B cells (Audzevich et al., 2017), and are preferentially supplied from the B cell precursors in the BM. Importantly, M-B cells are the major source of IL-10 in the BM after LPS treatment. Through the production of IL-10, M-B cells significantly boost EM by protecting hematopoietic progenitors from apoptosis and driving their myeloid-biased hematopoiesis, promoting the clearance of microbes in a cecal ligation and puncture (CLP) model. Collectively, M-B cells, a unique subset of B lymphocytes, contribute to the EM-mediated host defense against infections through their prominent production of IL-10.

## Results

### Increase of CD11b-expressing B cells in the BM during EM

**Figure 1.**
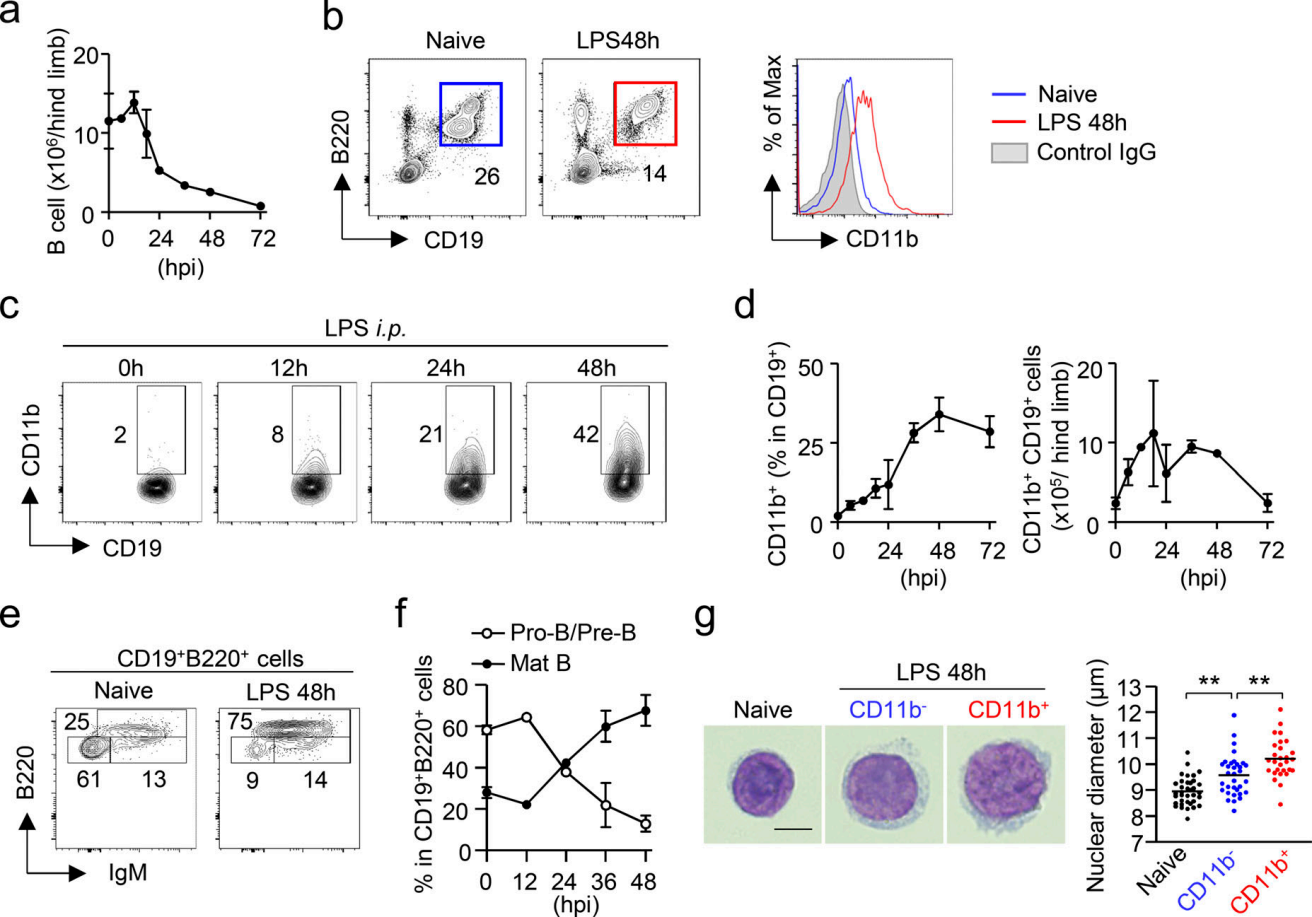
**CD11b-expressing B cells are increased in the BM during EM. (a)** Kinetic analysis of B cell numbers in the hind limb within 72 h after LPS injection (5 mg/kg). *n* = 3 for each time point. **(b)** Expression of CD11b on CD19^+^B220^+^ B cells in the BM before and 48 h after LPS treatment. **(c and d)** Frequencies and numbers of CD11b^+^ cells and CD19^+^ B cells in the BM after LPS treatment. Representative FCM plots are shown in panel c. *n* = 3 for each time point. **(e and f)** Kinetic analysis of mature B cells (CD19^+^B220^hi^IgM^+^) and pre B/pro B cells (CD19^+^B220^low^IgM^−^) in the BM before and after LPS treatment. Representative FCM plots for naive and 48 h after LPS treatment are shown in panel e. *n* = 3 per time point in each group. **(g)** Cytological images of mature B cells. CD19^+^B220^hi^IgM^+^ cells were obtained from the BM of naive mice, and CD11b^−^CD19^+^B220^hi^IgM^+^ and CD11b^−^CD19^+^B220^hi^IgM^+^ cells were obtained from the BM 48 h after LPS treatment. Cells were stained using Diff Quik. Diameters of nuclei for each population are shown in the right panel. *n* = 36 for naive, *n* = 33 for CD11b^−^ B cells, *n* = 28 for CD11b^+^ B cells. ** P < 0.01 (one-way ANOVA). Data are representative of two (a–d and g) or three (e and f) independent experiments (error bars, SD [a, d, and f]).

**Figure S1.**
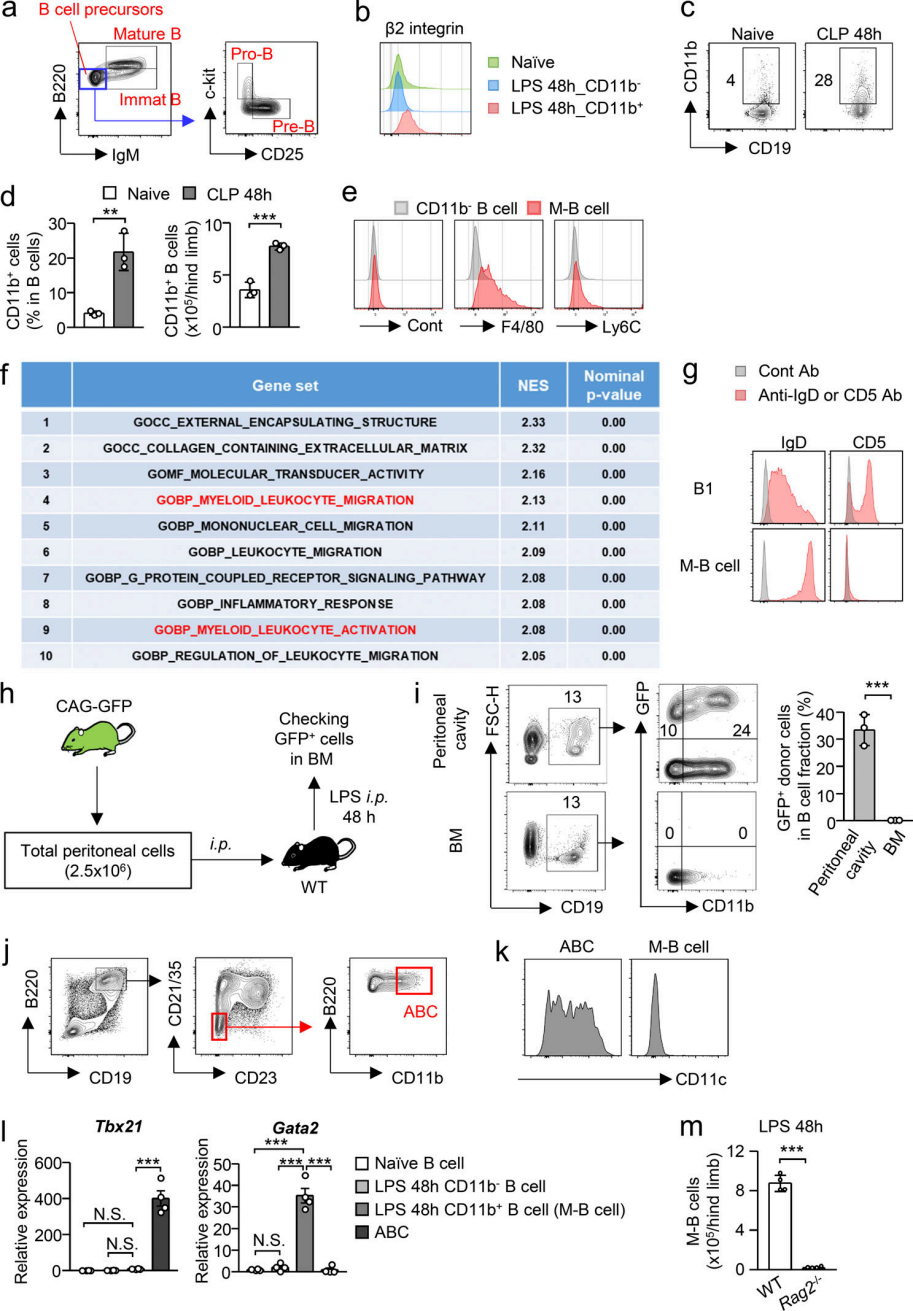
**M-B cells are distinct from other CD11b**^**+**^ **B cell subsets. (a)** Gating strategy of B cell lineages in the BM of naive WT C57BL/6 mice. **(b)** Expression of β2 integrin on B cells (CD19^+^B220^+^) obtained from the BM of naive WT mice and CD11b^−^ or CD11b^+^ B cells obtained from the BM of WT mice treated with LPS. **(c and d)** Expression of CD11b on B cells 48 h after CLP induction. Representative FCM plots are shown in panel c. Frequency and number of CD11b^+^ B cells are shown in panel d. *n* = 3 per group. **(e)** Expression of F4/80 and Ly6C on CD11b^−^ and CD11b^+^ B cells (M-B cells) obtained from the BM 48 h after CLP induction. **(f)** GSEA using RNA-sequencing data (FPKM+1, P < 0.05) with 1,572 ontology gene sets was performed and the top 10 gene sets are shown. Myeloid-related gene sets were shown in red. **(g)** Expression of IgD and CD5 on B1 in peritoneal cavity of naive mice and M-B cells. B1 cells were obtained from the peritoneal cavity of naive WT mice. **(h and i)** Tracing of peritoneal B1 cells in the BM after LPS treatment. EGFP-expressing peritoneal cells were transferred to the peritoneal cavity of WT recipient mice and migration of the cells to the BM was examined 48 h after LPS treatment. The experimental strategy is shown in panel h. *n* = 3 per group. **(j and k)** Phenotypical characterization of ABCs obtained from the spleen of 1.5-yr-old WT mice. Staining strategy to identify ABCs is shown in panel i and the expression of CD11c on ABCs obtained from aged mice and M-B cells obtained from the BM after LPS treatment is shown in panel k. **(l)** Expression of *Tbx21* and *Gata2* in ABCs, total B cells obtained from the BM of naive WT mice, CD11b^−^ and CD11b^+^ B cells (M-B cells) obtained from the BM of LPS-treated mice. Gene expression was evaluated by quantitative PCR. *n* = 4 per group. **(m)** Number of M-B cells in the BM of WT and *Rag2*^−/−^ mice 48 h after LPS treatment. *n* = 4 per group. N.S., not significant (P > 0.05), ** P < 0.01, *** P < 0.001 (Student’s *t* test [d, h, and l] or one-way ANOVA [l]). Data are representative of two independent experiments (a–g, i–k, and m) or from two independent experiments (l) (error bars, SD [d, i, and m] and SEM [l]). Symbols represent individual mice.

CD19^+^ B cells are abundant in the BM of naive mice (Fig. 1, a and b) and are composed of B220^hi^IgM^+^ mature B cells, B220^lo^IgM^+^ immature B cells, and B220^lo^IgM^−^ B cell precursors. B220^lo^IgM^−^ B cell precursors are further subdivided into CD25^+^ pre-B and c-kit^+^ pro-B cells (Fig. S1 a). We examined the quantitative alteration of CD19^+^ B cells in the BM during EM. To mimic an infection, we intraperitoneally injected LPS into C57BL/6J (WT) mice and found that the CD19^+^ B cell population was greatly reduced in the BM within 2 d after the injection (Fig. 1 a). On the contrary, the number of cells expressing CD11b, a pan-myeloid marker, and β2 integrin, which forms a heterodimer with CD11b, increased in the CD19^+^ B cell population (Fig. 1, b–d; and Fig. S1 b). A similar phenomenon was observed in the CLP model, a standard model for polymicrobial sepsis (Fig. S1, c and d). As the majority of the CD19^+^ B cell population was B220^hi^IgM^+^ mature B cells in the BM 48 h after LPS injection (Fig. 1, e and f), we isolated CD11b^+^ and CD11b^−^ cells from the mature B cell fraction at that time point and confirmed that CD11b^+^ B cells were morphologically lymphocytes, i.e., small cells with a higher nuclear-cytoplasmic ratio, although the nuclear size of CD11b^+^ B cells was slightly larger than that of CD11b^−^ B cells (Fig. 1 g).

### CD11b^+^ B cells generated during sepsis express myeloid markers and genes

**Figure 2.**
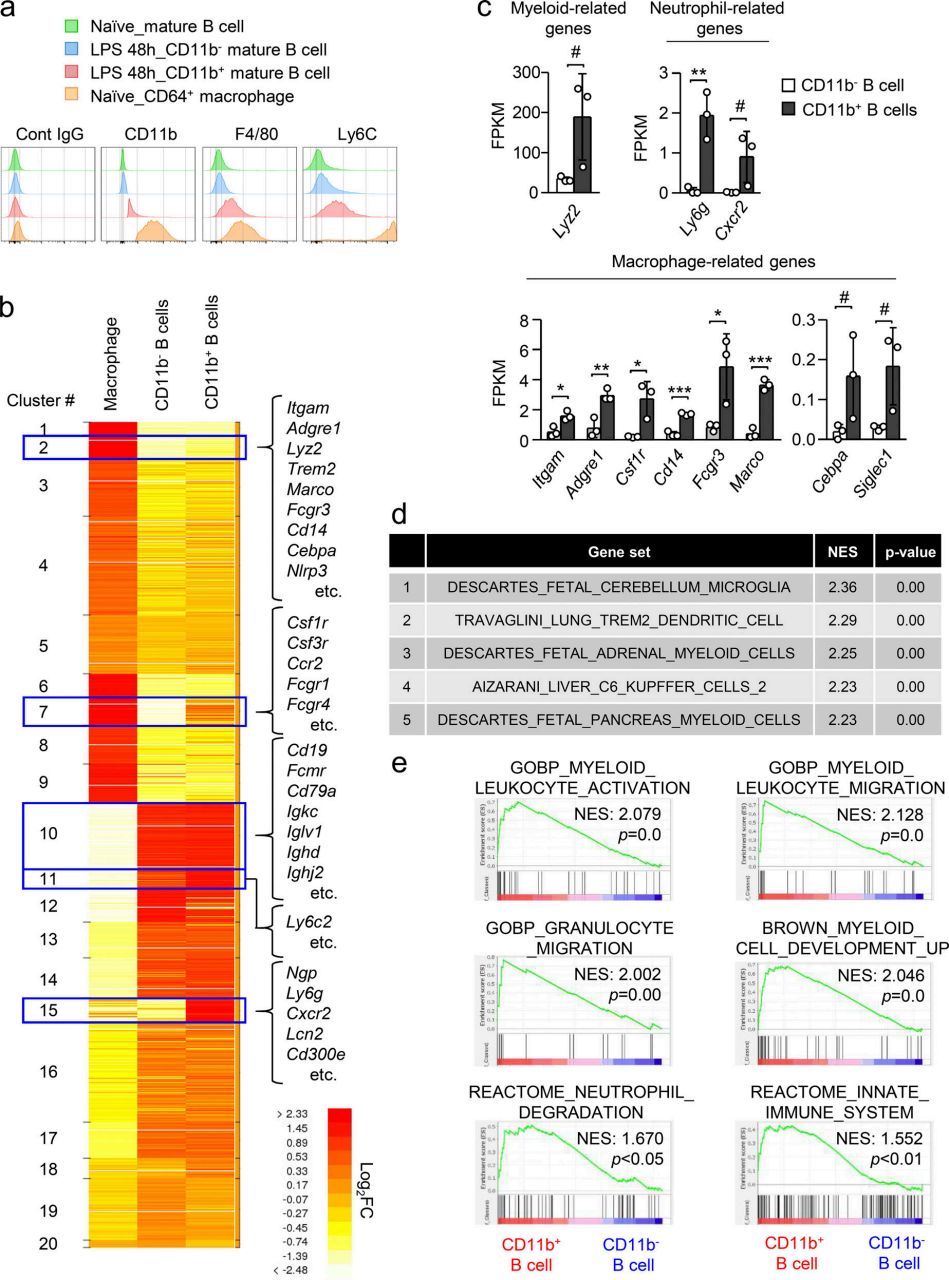
**CD11b**^**+**^ **B cells induced by sepsis express myeloid markers and genes. (a)** Expression of myeloid cell markers on the surface of mature B cells obtained from the BM before and 48 h after LPS treatment. **(b)** Heatmap of normalized count of RNA sequencing for CD11b^−^ B cells and CD11b^+^ B cells obtained from the BM 48 h after LPS treatment (5 mg/kg) and PMs (CD11b^+^F4/80^+^) obtained from naive mice. Data were shown as an average of three samples in each group. **(c)** FPKM values of myeloid-related genes. *n* = 3 each. **(d and e)** GSEA for RNA-sequencing data (FPKM+1, P < 0.05) of CD11b^−^ and CD11b^+^ B cells obtained from the BM 48 h after LPS treatment (5 mg/kg). Top five cell type signature gene sets enriched in CD11b^+^ B cells are shown in panel d and GSEA results for six gene sets that are related to myeloid activation, migration, function, and differentiation are shown in panel e. NES, normalized enrichment score. # P < 0.1, * P < 0.05, ** P < 0.01, *** P < 0.001 (*t* test [c]). Data are representative of two (b–e) or three (a) independent experiments (error bars, SD [c]).

Screening of 255 cell surface molecules revealed that CD11b^+^ B cells expressed other myeloid markers, such as F4/80 and/or Ly6C, compared with CD11b^−^ B cells (Fig. 2 a and Fig. S1 e). RNA-sequencing analyses revealed that multiple genes characteristic of myeloid cells, such as *Itgam*, *Adgre1*, *Lyz2*, *Csf1r*, *Ccr2*, *Cebpa*, *Ngp*, *Ly6g*, and *Cd300e*, were upregulated in CD11b^+^ B cells compared with CD11b^−^ B cells, although the expression levels of most myeloid genes in CD11b^+^ B cells were lower than in macrophages (Fig. 2, b and c). In addition, we performed gene set enrichment analysis (GSEA) using RNA-sequencing data of CD11b^+^ and CD11b^−^ B cells from the BM of LPS-treated mice with cell type signature gene sets (Fig. 2d) and ontology gene sets (Fig. S1 f), and again confirmed that multiple myeloid cell–associated genes were enriched in CD11b^+^ B cells (Fig. 2 d and Fig. S1 f). Consistently, GSEA using RNA-sequencing data of CD11b^+^ and CD11b^−^ B cells from the BM of LPS-treated mice showed that the signature genes for myeloid cell activation, migration, differentiation, and function were significantly enriched in CD11b^+^ B cells (Fig. 2 e). Based on these findings, the myeloid marker-bearing B cells are hereafter referred to as M-B cells.

B1 cells and ABCs are unique B cell subsets expressing CD11b. Although B1 cells are characterized by a lower expression level of IgD (Amendt et al., 2021), and a subpopulation of B1 cells (B1a) expresses CD5, most M-B cells express high levels of IgD and do not express CD5 (Fig. S1 g). We next collected cells from the peritoneal cavity of CAG (hybrid promoter consisting of CMV enhancer, chicken β-actin promoter, and rabbit β-globin intron)-EGFP mice, where B1 cells are abundant, and intraperitoneally injected the EGFP-expressing cells into WT mice. After LPS treatment, peritoneal B1 cells did not migrate to the BM, confirming that the M-B cells appear not to be B1 cells relocated from the peritoneal cavity (Fig. S1, h and i). ABCs were characterized as CD19^+^B220^+^CD21^−^CD23^−^CD11b^+^CD11c^+^ cells (Cancro, 2020; Fig. S1, j and k). However, M-B cells did not express CD11c (Fig. S1 k). In addition, M-B cells did not express *Tbx21*, which is preferentially expressed by ABCs (Cancro, 2020; Fig. S1 l). Instead, M-B cells expressed *Gata2* (Fig. S1 l). Furthermore, pro-B cells expressing CD11b have been reported as a precursor of peritoneal macrophages (PMs; Audzevich et al., 2017). In *Rag2*^*−/−*^ mice, pro-B cells and pro-B cell–derived PMs exist (Audzevich et al., 2017), whereas M-B cells were completely absent (Fig. S1 m), suggesting that they are distinct populations. Collectively, M-B cells appeared to be a unique subset of B cells, which are different from B1 cells, ABCs, and CD11b^+^ pro-B cells.

### M-B cells are mainly derived from B cell precursors in the BM

**Figure 3.**
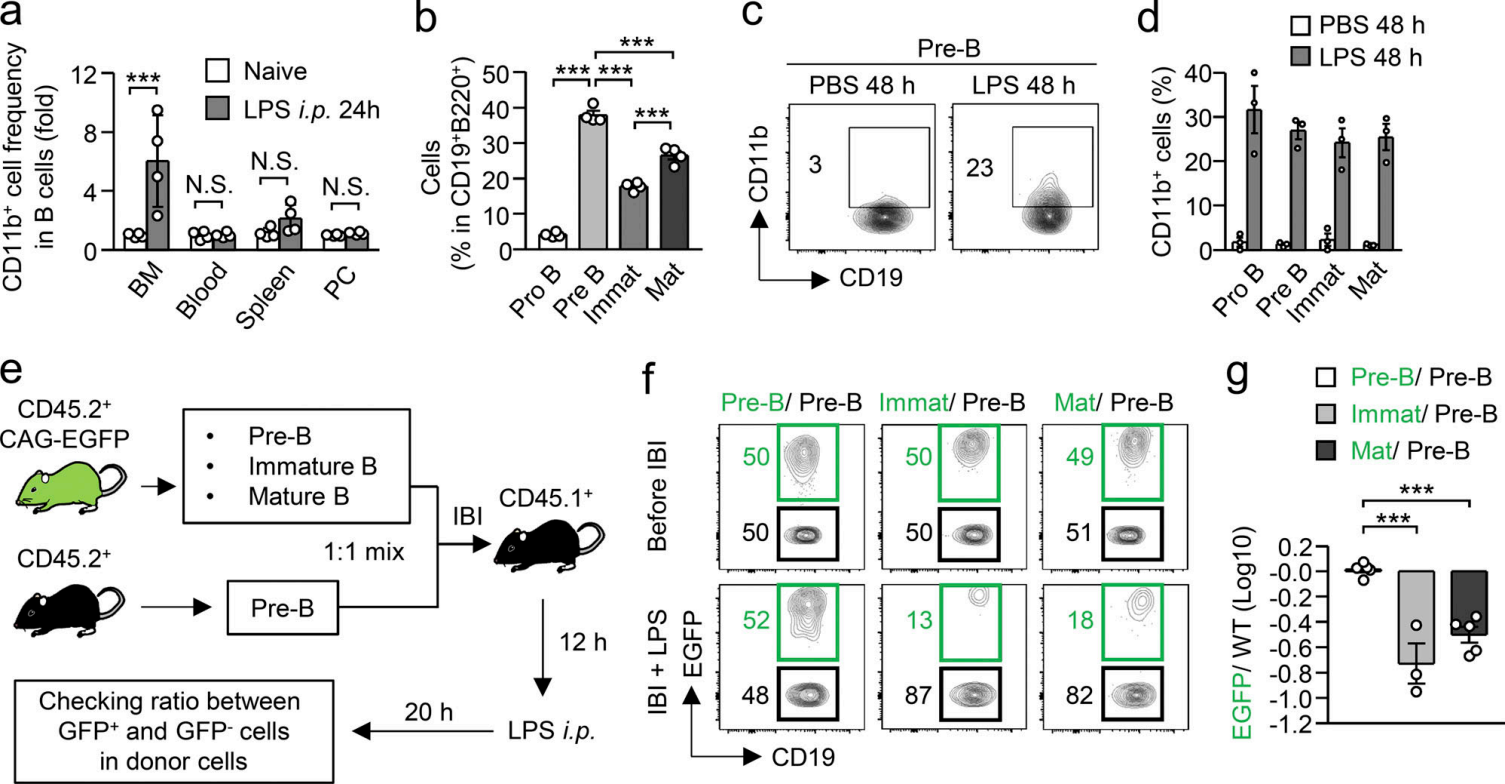
**M-B cells are mainly derived from B cell precursors. (a)** Fold change of frequency of CD11b^+^ cells in CD19^+^B220^+^ B cells in the BM, blood, spleen, and peritoneal cavity (PC) 24 h after LPS treatment. *n* = 4 each. **(b)** Cell proportions of B cell lineages such as pro-B, pre-B, immature (Immat), and mature (Mat) B cells in the BM of naive mice. *n* = 4 each. **(c and d)** Capacity of B cell lineages to upregulate CD11b upon LPS treatment. Pre-B, pro-B, Immat B, and Mat B cells were isolated from the BM of naive CD45.2^+^ mice and were directly injected into the tibias of CD45.1^+^ recipient mice. 12 h after the IBI of cells, LPS (5 mg/kg) was administered intraperitoneally and CD11b expression by the BM-resident donor cells was examined as shown in Fig. S2 c. Frequencies of CD11b^+^ cells in each donor cell at 48 h after LPS treatment are shown in panel d. *n* = 3 each. **(e–g)** Capacity of B cell lineages to stay in the BM after LPS treatment. Pre-B, Immat B, or Mat B cells were obtained from CD45.2^+^ CAG-EGFP mice and were mixed at a 1:1 ratio with Pre-B cells obtained from CD45.2^+^ WT mice. The mixed cells were injected into the tibias of CD45.1^+^ recipient mice, and the mice were treated with LPS 12 h after IBI as shown in panel e. The ratios between EGFP^−^ and EGFP^+^ cells in BM-resident donor cells are shown in panel g. *n* = 5 for Pre-B/Pre-B and Mat/Pre-B and *n* = 3 for Immat/Pre-B. N.S., not significant (P > 0.05). * P < 0.05, *** P < 0.001 (one-way ANOVA [a, b, and g]). Data are representative of two (a) or three (c and f) independent experiments or from three (b, d, and g) independent experiments (error bars, SD [a] and SEM [b, d, and g]).

**Figure S2.**
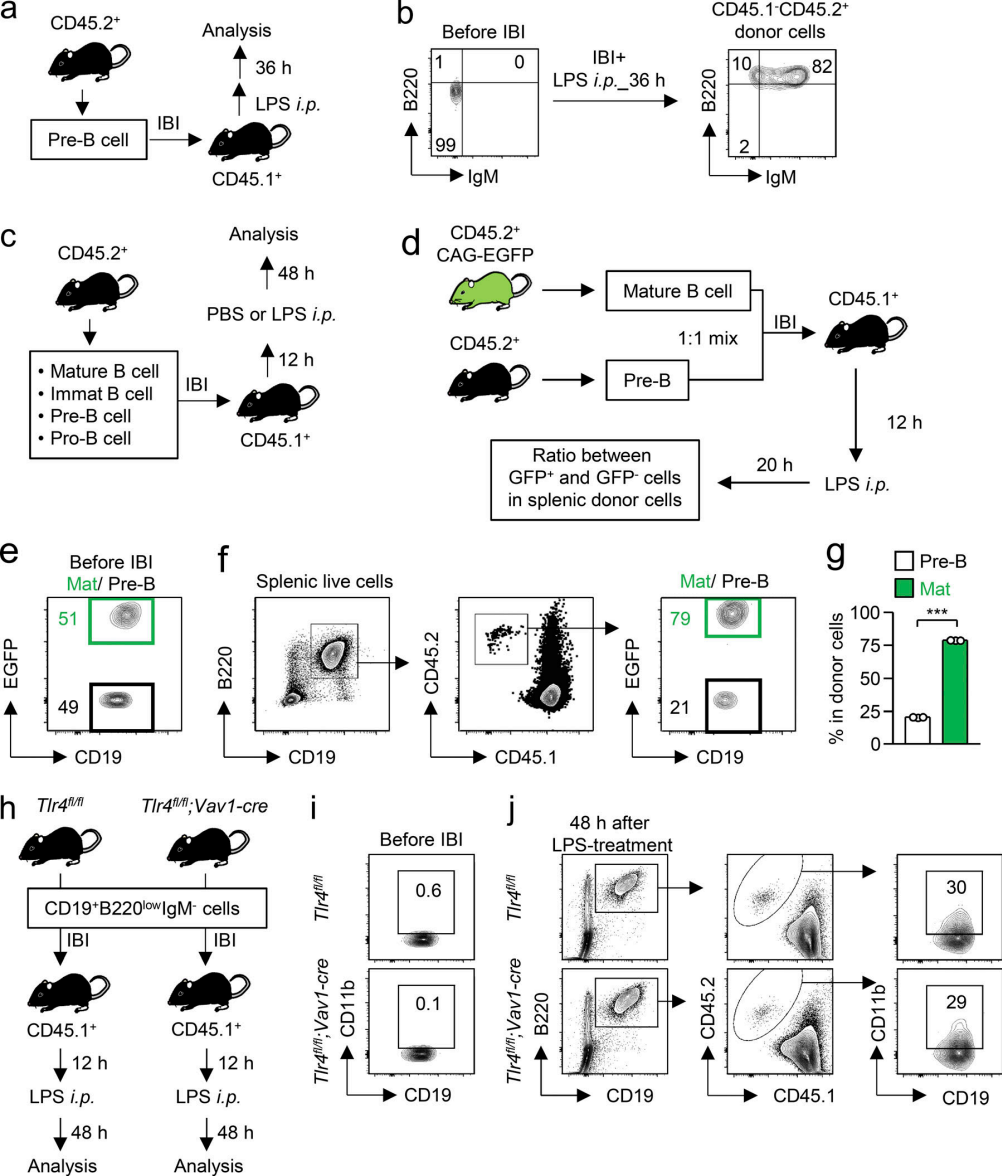
**Intra-BM transplantation of B cell lineages. (a and b)** Differentiation of mature B cells from pre-B cells after LPS treatment. Pre-B cells obtained from WT mice were transplanted into the tibias of CD45.1^+^ mice and the donor-derived cells in the BM of recipient mice were analyzed 36 h after intraperitoneal LPS injection (5 mg/kg). Experimental strategy is shown in panel a. Representative FCM plots are shown in panel b. Data are representative of two independent experiments. **(c)** Experimental strategy for Fig. 3, c and d. Pre-B, pro-B, immature B, or mature B cells obtained from WT mice were transplanted into the tibias of CD45.1^+^ mice. LPS (5 mg/kg) was intraperitoneally injected into the recipient mice 12 h after IBI and the donor-derived cells were analyzed 48 h after LPS treatment. **(d–g)** Migration capacity of mature B cells and pre-B cells from the BM to the periphery after LPS treatment. Experimental strategy is shown in panel d. Mature B cells obtained from the BM of CAG-EGFP mice were mixed at a 1:1 ratio with pre-B cells obtained from WT mice (e). These mixed cells were transplanted into the BM of CD45.1^+^ recipient mice. LPS (5 mg/kg) was intraperitoneally injected into the recipient mice 12 h after IBI and the donor-derived cells were analyzed 20 h after LPS treatment (f and g). *n* = 3 per group. **(h–j)** Importance of TLR4 expressed by B cell precursors to generate M-B cells. B cell precursors (CD19^+^B220^low^IgM^−^) obtained from the BM of *Tlr4*^*fl/fl*^ and *Tlr4*^*fl/fl*^*; Vav1-cre* mice were transplanted into the BM of CD45.1-expressing recipient mice. The mice were treated with LPS (5 mg/kg) 12 h after transplantation. Before (i) and 48 h after (j) LPS treatment, CD11b expression on the donor B cells was examined by FACS. Experimental strategy is shown in panel h. *** P < 0.001 (Student’s *t* test). Data are representative of two independent experiments (error bars, SD).

We next asked how M-B cells develop in the BM. After LPS treatment, the frequency of CD11b^+^ B cells significantly increased early in the BM but not in the blood, spleen, or peritoneal cavity (Fig. 3 a), suggesting that M-B cells are derived from B cell precursors that represent about half of CD19^+^B220^+^ cells in the BM of naive mice (Fig. 1, e and f; and Fig. 3 b). Upon LPS treatment, the number of B cell precursors dramatically decreased and the number of mature B cells conversely increased (Fig. 1, e and f), implying the immediate differentiation of B cell precursors into mature B cells. Indeed, pre-B cells that had been transplanted into the BM differentiated into mature B cells within 36 h after LPS treatment (Fig. S2, a and b). To identify the main source of M-B cells, mature, immature, pre-, or pro-B cells isolated from CD45.2^+^, WT mice were transplanted into the BM of CD45.1^+^ B6.SJL-ptprca (B6.SJL) mice, and CD11b expression by the donor-derived B cells was examined 48 h after PBS or LPS injection (Fig. 3, c and d; and Fig. S2 c). The expression of CD11b was low after PBS treatment of mice transplanted with any of the donor cells (Fig. 3, c and d). However, upon LPS treatment, CD11b-expressing M-B cells emerged at similar frequencies in each population of donor cell–derived B cells (Fig. 3, c and d), which suggests that M-B cells are derived from all stages of BM-B cell lineages after LPS treatment. We next examined the retention capacity of mature, immature, and pre-B cells after LPS treatment. A 1:1 mixture of pre-B cells from CD45.2^+^ WT mice and pre-B cells, immature B cells, or mature B cells from CD45.2^+^ CAG-EGFP mice was injected into the BM of CD45.1^+^ B6.SJL mice (intra BM injection, IBI), and the ratio of EGFP^+^ and WT donor cells in the BM was compared 20 h after LPS treatment (Fig. 3 e). As a control, a 1:1 mixture of EGFP^+^ pre-B cells and EGFP^−^ pre-B cells was equally retained in the BM after IBI and LPS treatment (Fig. 3, f and g). In contrast, the frequency of EGFP^+^ immature or mature B cells was dramatically decreased in the BM compared with EGFP^−^ pre-B cells (Fig. 3, f and g). In contrast, when EGFP^−^ pre-B cells and EGFP^+^ mature B cells were mixed at 1:1 and transplanted into CD45.1^+^ B6.SJL mice (Fig. S2, d and e), the frequency of EGFP^+^ mature B cells became significantly higher than that of EGFP^−^ pre-B cells in the spleen after LPS treatment (Fig. S2, f and g), which suggests that the capacity of pre-B cells to stay in the BM was much higher than mature and immature B cells after LPS treatment. Considering that immature and mature B cells rapidly leave the BM after LPS administration, B cell precursors containing pre- and pro-B cells seem to be a major source of M-B cells in the BM.

To examine the mechanism of M-B cell induction, we tested whether TLR4-mediated signaling directly induces M-B cell differentiation from B cell precursors. We found that both TLR4-sufficient and TLR4-deficient pre-/pro-B cells transplanted into the BM of recipient mice also clearly express CD11b after LPS treatment (Fig. S2, h–j), suggesting that TLR4-mediated signaling on B cells is not essential for M-B cell generation. Rather, inflammatory cytokines indirectly upregulated in other cells by LPS likely induce M-B cells.

### M-B cells are the source of IL-10 in the BM of LPS-treated mice

**Figure 4.**
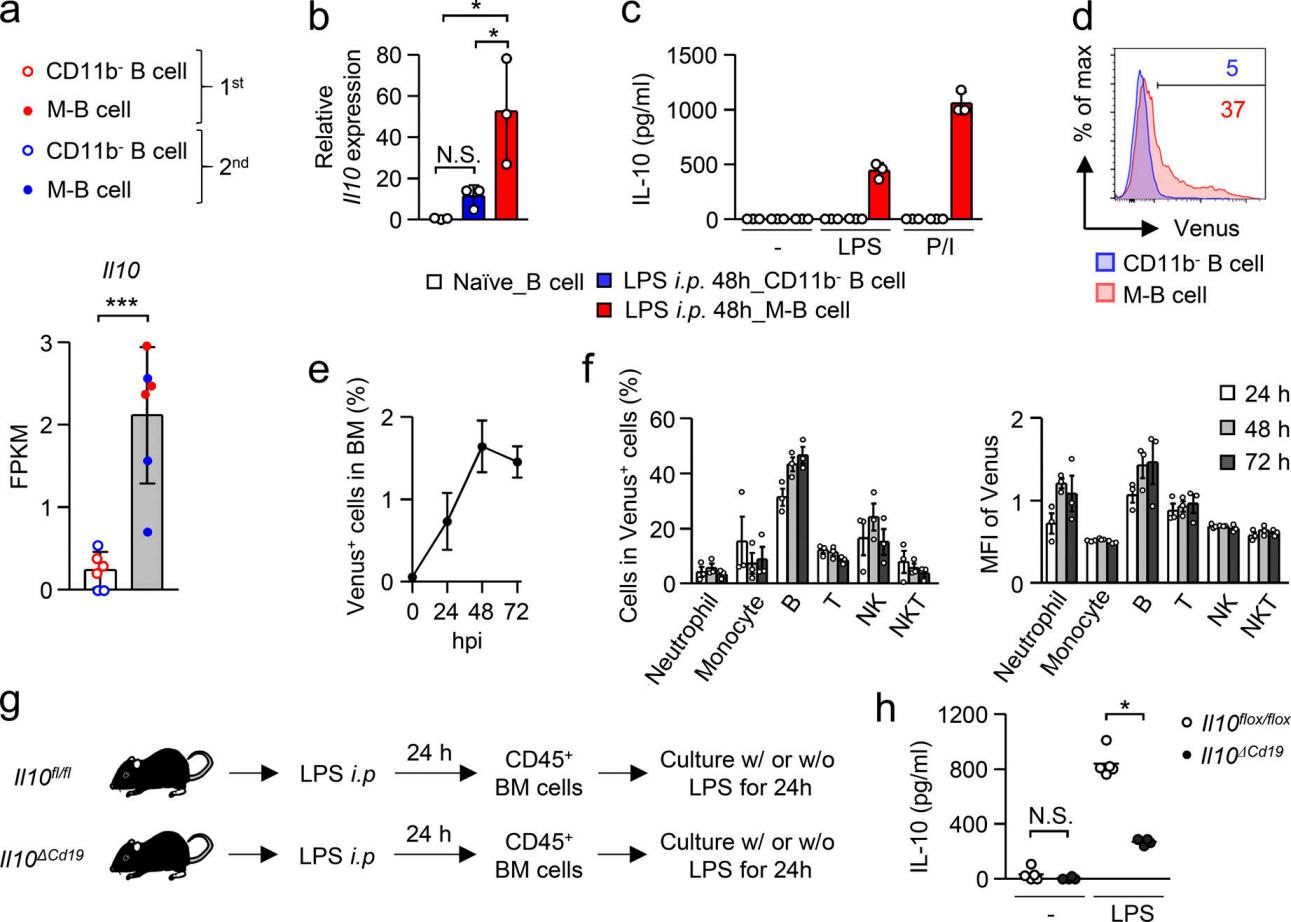
**M-B cells are a major source of IL-10 in the BM of LPS-treated mice. (a)** Expression of *Il10* in CD11b^−^ B cells and M-B cells obtained from the BM 48 h after LPS treatment (5 mg/kg). RNA sequencing was performed twice and FPKM values of *Il10* from each trial (first and second, *n* = 3 of each) are shown. **(b)** Expression of *Il10* in total B cells obtained from the BM of naive mice and CD11b^−^ and M-B cells obtained from mice treated with LPS. Gene expression of *Il10* was assessed by real-time PCR. *n* = 3 each. **(c)** IL-10 secretion capacity of B cells. Total B cells or CD11b^−^ and M-B cells were obtained from naive or LPS-treated mice, respectively. The cells were cultured in the absence or presence of LPS (100 ng/ml) or PMA (20 ng/ml) and ionomycin (250 ng/ml) for 24 h and the supernatants were subjected to ELISA analysis to determine levels of IL-10 secretion. *n* = 3 each. **(d)** Evaluation of *Il10* expression using *Il10*-Venus reporter mice. *Il10*-Venus reporter mice were treated with LPS (5 mg/kg) and Venus expression in CD11b^−^ and M-B cells was evaluated in the BM by FCM analysis. **(e)** Ratio of *Il10*-Venus^+^ cells in BM cells after LPS treatment. *n* = 3 for each time point. **(f)** Composition of Venus^+^ cells in BM cells after LPS treatment. The frequency of neutrophils (CD11b^+^Ly6C^+^Ly6G^+^), monocytes (CD11b^+^Ly6C^high^Ly6G^−^), B cells (CD19^+^B220^+^), T cells (CD3^+^NK1.1^−^), natural killer (NK) cells (CD3^−^NK1.1^+^), and NKT cells (CD3^+^NK1.1^+^) in Venus^+^ cells is shown at 24, 48, and 72 h after LPS treatment. *n* = 3 for each time point. **(g and h)** Impact of *Il10* deficiency in B cells on IL-10 production by BM cells. *Il10*^*ΔCd19*^ mice (*n* = 4) and *Il10*^*fl/fl*^ mice (*n* = 5) were treated with LPS (5 mg/kg). 24 h after that, CD45^+^ BM cells were cultured in the absence or presence of LPS (100 ng/ml) for 24 h. The supernatants were collected and subjected to ELISA analysis to determine levels of IL-10 secretion. N.S., not significant (P > 0.05), * P < 0.05, *** P < 0.001 (Student *t* test [a], one-way ANOVA [b and h]). Data are representative of two (b–d) independent experiments or from two (a and h) or three (a, e, and f) independent experiments (error bars, SD [a–c] and SEM [e and f]).

**Figure S3.**
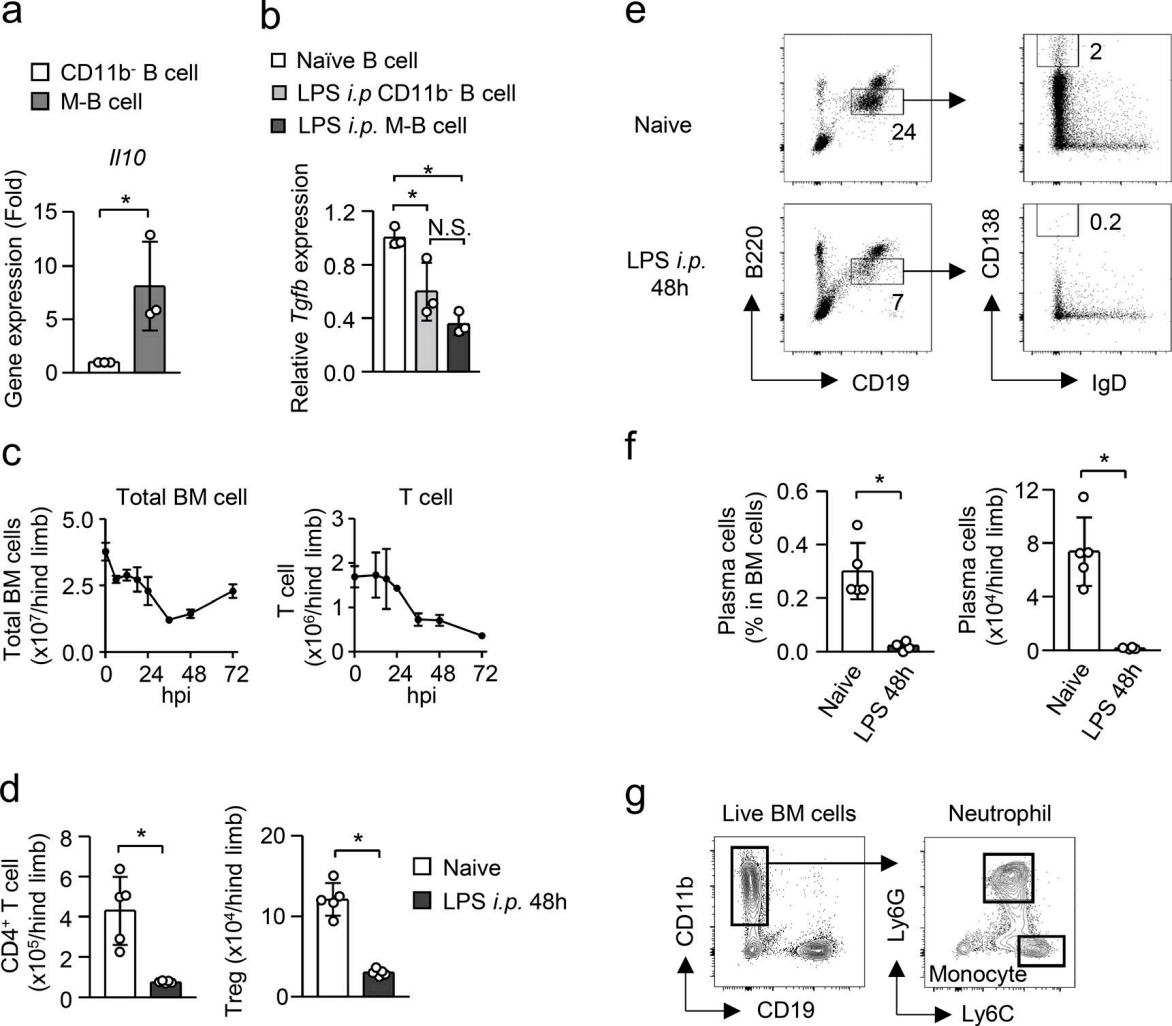
**Disappearance of Tregs and plasma cells in the BM after LPS treatment. (a)** Evaluation of *Il10* gene expression in CD11b^−^ B cells and in M-B cells obtained from the BM of WT mice 48 h after CLP induction by quantitative PCR analysis. The fold change of *Il10* expression between the two groups is shown. **(b)** Gene expression of *Tgfb* in total B cells obtained from the BM of naive mice and CD11b^−^ and M-B cells obtained from the BM after LPS treatment (5 mg/kg). Gene expression was assessed by quantitative PCR analysis. *n* = 3 per group. **(c)** Kinetic analysis of cell numbers for total BM cells and T cells in the hind limbs of WT mice after LPS treatment. *n* = 3 per group. **(d)** Number of CD4^+^ T cells and Treg cells (CD3^+^CD4^+^Foxp3^+^) in the BM before and 48 h after intraperitoneal injection of LPS (5 mg/kg). **(e and f)** Number and frequency of plasma cells in the BM before and after intraperitoneal injection of LPS (5 mg/kg). *n* = 5 for naive and *n* = 4 for 48 h after LPS-treatment. **(g)** Gating strategy of neutrophils and monocytes. Monocytes and neutrophils were defined as CD11b^+^Ly6C^hi^Ly6G^−^ and CD11b^+^Ly6C^+^Ly6G^+^, respectively. N.S., not significant (P > 0.05), * P < 0 05 (Student’s *t* test [a, d, and f] or one-way ANOVA [b]). Data are representative of two independent experiments (error bars, SD [a–d and f]). Symbols represent individual mice.

We then functionally characterized M-B cells. To find the functional molecules of M-B cells, we performed RNA sequencing of CD11b^−^ and CD11b^+^ B cells obtained from the BM 48 h after LPS treatment, and *Il10* was identified as an upregulated gene of M-B cells (Fig. 4 a). The upregulation of *Il10* on M-B cells was confirmed by quantitative PCR (Fig. 4 b). Indeed, when total B cells from the BM of naive mice or CD11b^−^ B cells or M-B cells from the BM of LPS-treated mice were cultured with LPS or PMA and ionomycin, only M-B cells secreted IL-10 (Fig. 4 c). Analysis using *Il10*-Venus reporter mice, which have been used to detect IL-10 expression in various cell types including monocytes, macrophages, dendritic cells, T cells, and plasma blasts (Atarashi et al., 2011; Hayashi et al., 2013; Matsumoto et al., 2014; Morhardt et al., 2019; Ochi et al., 2016; Ohyagi et al., 2013), also showed a clear contrast in IL-10 expression between M-B cells and CD11b^−^ B cells after LPS treatment (Fig. 4 d). The upregulation of *Il10* in M-B cells was also observed in the BM of CLP-induced mice (Fig. S3 a). In addition to IL-10, TGF-β has been suggested to function as a regulatory factor expressed by regulatory B cells (Rincon-Arevalo et al., 2016), but expression of the *Tgfb* gene was not upregulated in M-B cells (Fig. S3 b).

Analysis using *Il10*-Venus reporter mice showed an increase of *Il10*-expressing cells in the BM of LPS-treated mice (Fig. 4 e). Importantly, B cells were the largest population among the *Il10*-expressing cells, and the B cell expression level of *Il10* was the highest among the *Il10*-expressing populations (Fig. 4 f). Tregs and plasma cells are possible sources of IL-10 in the BM under naive conditions or after BM transplantation (Fujisaki et al., 2011; Meng et al., 2019). However, the number of Tregs was strongly reduced (Fig. S3, c and d) and plasma cells disappeared (Fig. S3, e and f) in the BM after LPS treatment as previously reported in systemic *Toxoplasma gondii* infections (Glatman Zaretsky et al., 2017). To further evaluate the importance of B cells as a major source of IL-10 in the BM, we cultured total leukocytes from the BM of mice lacking *Il10* in B cells (*Cd19-Cre:Il10*^*fl/fl*^ mice, hereafter denoted as *Il10*^*ΔCd19*^) or their littermate controls (*Il10*^*fl/fl*^ mice) in the presence of LPS (Fig. 4 g). As expected, the lack of *Il10* in B cells strongly decreased IL-10 secretion from the total BM leukocytes (Fig. 4 h). Considering that IL-10–producing B cells are M-B cells rather than CD11b^−^ B cells in the BM after LPS treatment (Fig. 4, a–d), we conclude that M-B cells are the major source of IL-10 in the BM during LPS-induced EM.

### B cell–derived IL-10 boosts EM

**Figure 5.**
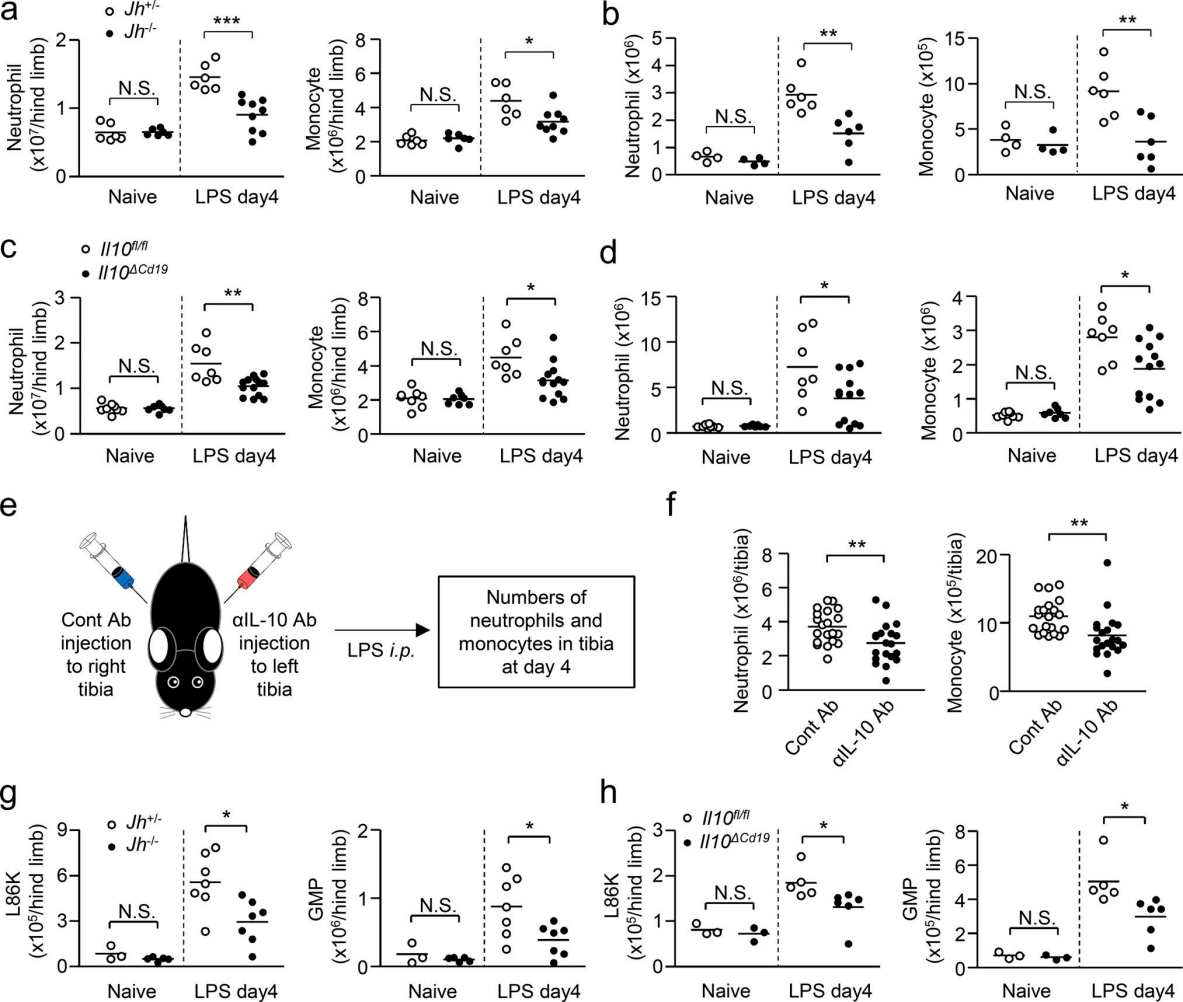
**B cell–derived IL-10 enhances EM. (a and b)** EM in mice lacking B cells after LPS treatment. *Jh*^*−/−*^ mice and their littermate control *Jh^+/^*^*−*^ mice received intraperitoneal injections of LPS (5 mg/kg). The numbers of neutrophils (CD11b^+^Ly6C^+^ Ly6G^+^) and monocytes (CD11b^+^Ly6C^hi^ Ly6G^−^) were examined in the BM (a) and spleen (b) 4 d after LPS treatment. *n* = 6 for naive *Jh*^−/−^, naive *Jh*^+/−^, and LPS-treated *Jh*^+/−^, *n* = 9 for LPS-treated *Jh*^−/−^. **(c and d)** EM in mice lacking *Il10* selectively in B cells after LPS treatment. *Il10*^*ΔCd19*^ mice and *Il10*^*fl/fl*^ mice received intraperitoneal injections of LPS (5 mg/kg). The numbers of neutrophils and monocytes were examined in the BM (c) and spleen (d) 4 d after LPS treatment. *n* = 8 for naive *Il10*^*fl/fl*^, *n* = 7 for naive *Il10*^*ΔCd19*^ and LPS-treated *Il10*^*fl/fl*^, *n* = 13 for LPS-treated *Il10*^*ΔCd19*^. **(e and f)** Impact of local IL-10 on EM. An anti–IL-10 neutralizing antibody or its isotype control (1 μg each) was injected into the BM of the left and right tibia, respectively. LPS (5 mg/kg) was then injected into the mice, and the numbers of neutrophils and monocytes in the BM of the tibias were examined 4 d after LPS treatment. Experimental strategy is shown in panel e. *n* = 20 per group. **(g and h)** Numbers of hematopoietic progenitors in mice lacking B cells or IL-10 production in B cells after LPS treatment. LPS (5 mg/kg) was intraperitoneally injected into *Jh*^−/−^ and *Jh*^+/−^ mice (g) or into *Il10*^*ΔCd19*^ and *Il10*^*fl/fl*^ mice (h), and the numbers of L86Ks and GMPs were examined 3 d later. *N* = 3 for *Jh*^+/−^, *Jh*^−/−^, *Il10*^*fl/fl*^, and *Il10*^*ΔCd19*^ mice in naive conditions, *n* = 7 for *Jh*^+/−^ and *Jh*^−/−^ mice after LPS treatment, *n* = 5 and *n* = 6 for *Il10*^*fl/fl*^ and *Il10*^*ΔCd19*^ mice, respectively, after LPS-treatment. N.S., not significant (P > 0.05), * P < 0.05, ** P < 0.01, *** P < 0.001 (Student’s *t* test [a–d and f–h]). Data are from two (g and h), three (a–d) or five (f) independent experiments. Symbols represent individual mice.

**Figure S4.**
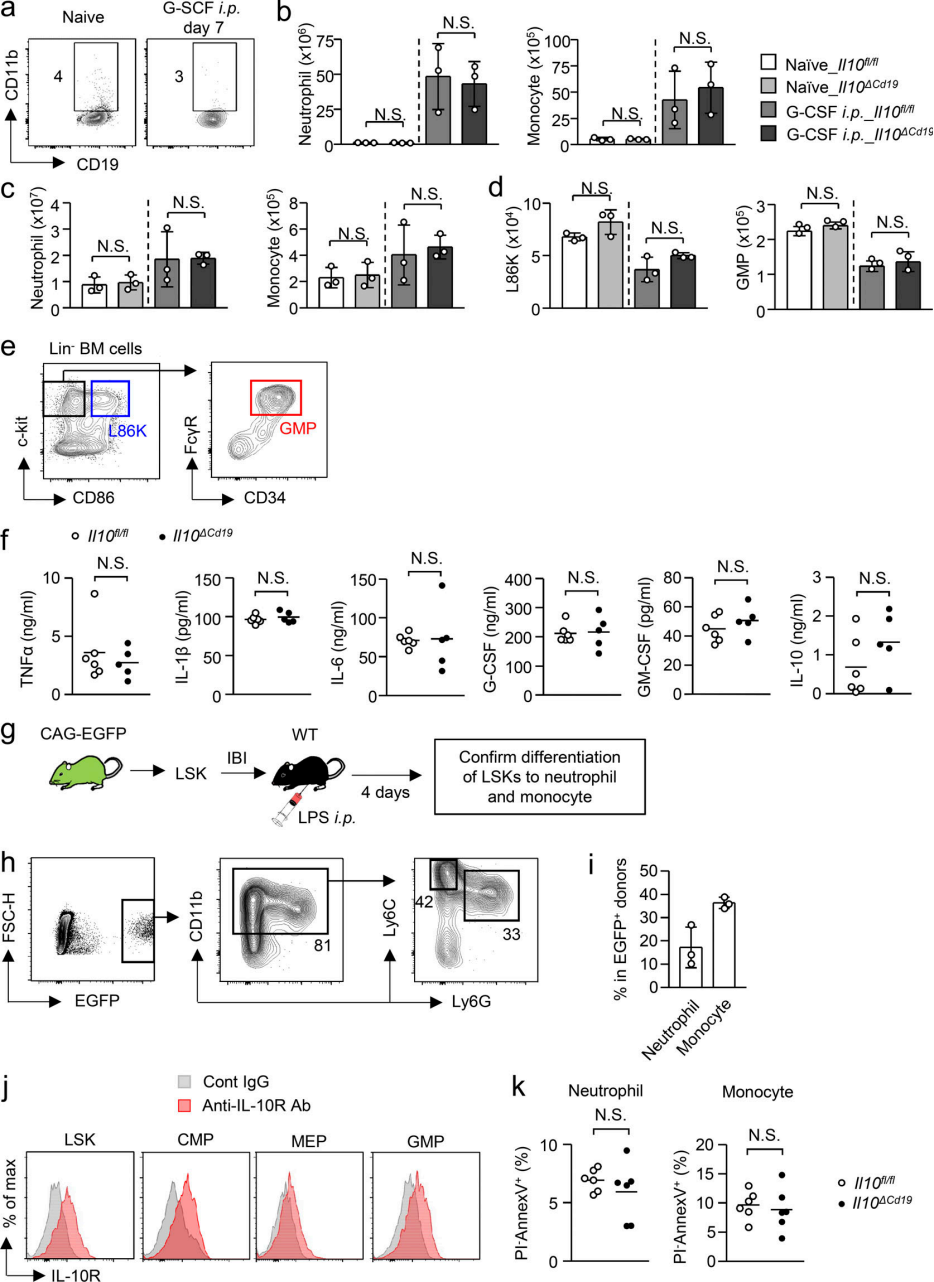
**Impact of B cell–derived IL-10 in cytokine production and apoptosis of myeloid cells. (a–d)** Requirement of B cell–derived IL-10 in G-CSF–induced myelopoiesis. *Il10*^*fl/fl*^ and *Il10*^*ΔCd19*^ mice were treated daily with G-CSF (5 μg/mouse) for 7 d and the numbers of neutrophils and monocytes in the spleen (b) and BM (c) and L86K cells and GMPs in the BM (d) were counted. The frequencies of CD11b^+^ B cells in the BM of *Il10*^*fl/fl*^ and *Il10*^*ΔCd19*^ mice are shown in panel a. *n* = 3 per group. **(e)** Gating strategy of L86K and GMP. **(f)** Serum cytokines and growth factors in *Il10*^*fl/fl*^ and *Il10*^*ΔCd19*^ mice 2 h after peritoneal administration of LPS (5 mg/kg). Levels of systemic cytokines and growth factors were evaluated by ELISA analysis. *n* = 6 for *Il10*^*fl/fl*^ and *n* = 5 for *Il10*^*ΔCd19*^. **(g–i)** Differentiation of LSKs to myeloid cells within 4 d after LPS treatment. LSKs obtained from EGFP-expressing mice were transferred into the tibias of WT mice and LPS (5 mg/kg) was injected. The differentiation of donor-derived cells was examined by FCM 4 d after LPS treatment. Experimental strategy and gating strategy are shown in panels g and h, respectively. *n* = 3 per group. **(j)** Expression of IL-10 receptor by hematopoietic progenitors in the BM of naive mice. CMP, common myeloid progenitor; MEP, megakaryocyte-erythrocyte progenitor. **(k)** Ratio of apoptotic cells (Annexin V^+^ PI^−^) in neutrophils and monocytes 48 h after LPS treatment. *n* = 6 per group. N.S., not significant (P > 0.05; Student’s *t* test [b–d, f, and k]). Data are from two independent experiments (error bars, SD [b–d and i]). Symbols represent individual mice.

To evaluate the importance of B cells in the induction of EM, we determined the number of CD11b^+^Ly6C^+^Ly6G^+^ neutrophils and CD11b^+^Ly6C^high^Ly6G^−^ monocytes (Fig. S3 g) in the BM and spleen of *Jh*^−/−^ mice, which lack B lymphocytes due to a deletion of *J*_*h*_ gene segments of the Ig heavy chain locus, and their littermate controls (*Jh*^+/−^ mice) before and 4 d after LPS treatment, the peak of neutrophil and monocyte expansion in the BM (Fig. 5, a and b). The numbers of neutrophils and monocytes in the BM and spleen were comparable between *Jh*^−/−^ and *Jh*^+/−^ mice in naive conditions. Upon LPS treatment, the numbers of those myeloid cells greatly increased in the BM and spleen of control *Jh*^+/−^ mice, whereas they were significantly attenuated in *Jh*^−/−^ mice (Fig. 5, a and b), which suggests the importance of B cells for inducing effective EM. As M-B cells are the source of IL-10 in B cells of the BM of LPS-treated mice (Fig. 4), we next examined the impact of M-B cell–derived IL-10 in EM by injecting LPS into B cell–specific IL-10–deficient (*Il10*^*ΔCd19*^) mice and into their littermate control *Il10*^*fl/fl*^ mice. In naive conditions, there was no significant difference in the numbers of neutrophils and monocytes between the two groups of mice (Fig. 5, c and d). However, as in *Jh*^−/−^ mice, LPS-induced myeloid cell generation was significantly reduced in *Il10*^*ΔCd19*^ mice (Fig. 5, c and d), suggesting the importance of M-B cell–derived IL-10 for inducing effective EM. On the other hand, during G-CSF–induced myelopoiesis, CD11b^+^ B cells did not emerge in the BM (Fig. S4 a) and an IL-10 deficiency in B cells did not alter the numbers of myeloid cells and their progenitors in the BM or spleen (Fig. S4, b–d), which suggests that the contribution of M-B cells in myelopoiesis is limited to situations of infection/sepsis.

To evaluate the importance of local IL-10 production in the BM, we injected a neutralizing antibody for IL-10 or a control antibody into the BM of the left and right tibia, respectively, and examined the numbers of neutrophils and monocytes in each tibia 4 d after LPS treatment (Fig. 5 e). Importantly, the local blockade of IL-10 significantly decreased myelopoiesis (Fig. 5 f), suggesting that local IL-10, predominantly supplied by M-B cells, contributes to the enhancement of EM.

### B cell–derived IL-10 protects hematopoietic progenitors from apoptosis in EM

Neutrophils and monocytes are generated from hematopoietic progenitors. We next examined whether B cells or B cell–derived IL-10 affects the numbers of hematopoietic progenitors during EM. In this context, we recently reported that Sca-1 is not a reliable marker to distinguish myeloid progenitors from hematopoietic stem progenitor cells (Lin^−^Sca-1^+^c-kit^+^, LSK) during infection and inflammation because Lin^−^Sca-1^−^c-kit^+^ (LK) cells containing myeloid progenitors gain Sca-1 expression (Kanayama et al., 2020). We identified CD86 as a reliable marker instead of Sca-1 (Kanayama et al., 2020). Using CD86, we identified LSK as L86K (Fig. S4 e). In agreement with the numbers of neutrophils and monocytes, the numbers of L86K and granulocyte-monocyte progenitors (GMPs) were significantly reduced in the absence of B cells and B cell–derived IL-10 after LPS treatment (Fig. 5, g and h). Given that M-B cells are the source of IL-10 in B cells of LPS-treated mice (Fig. 4), M-B cell–derived IL-10 acts on hematopoietic progenitors to enhance EM.

**Figure 6.**
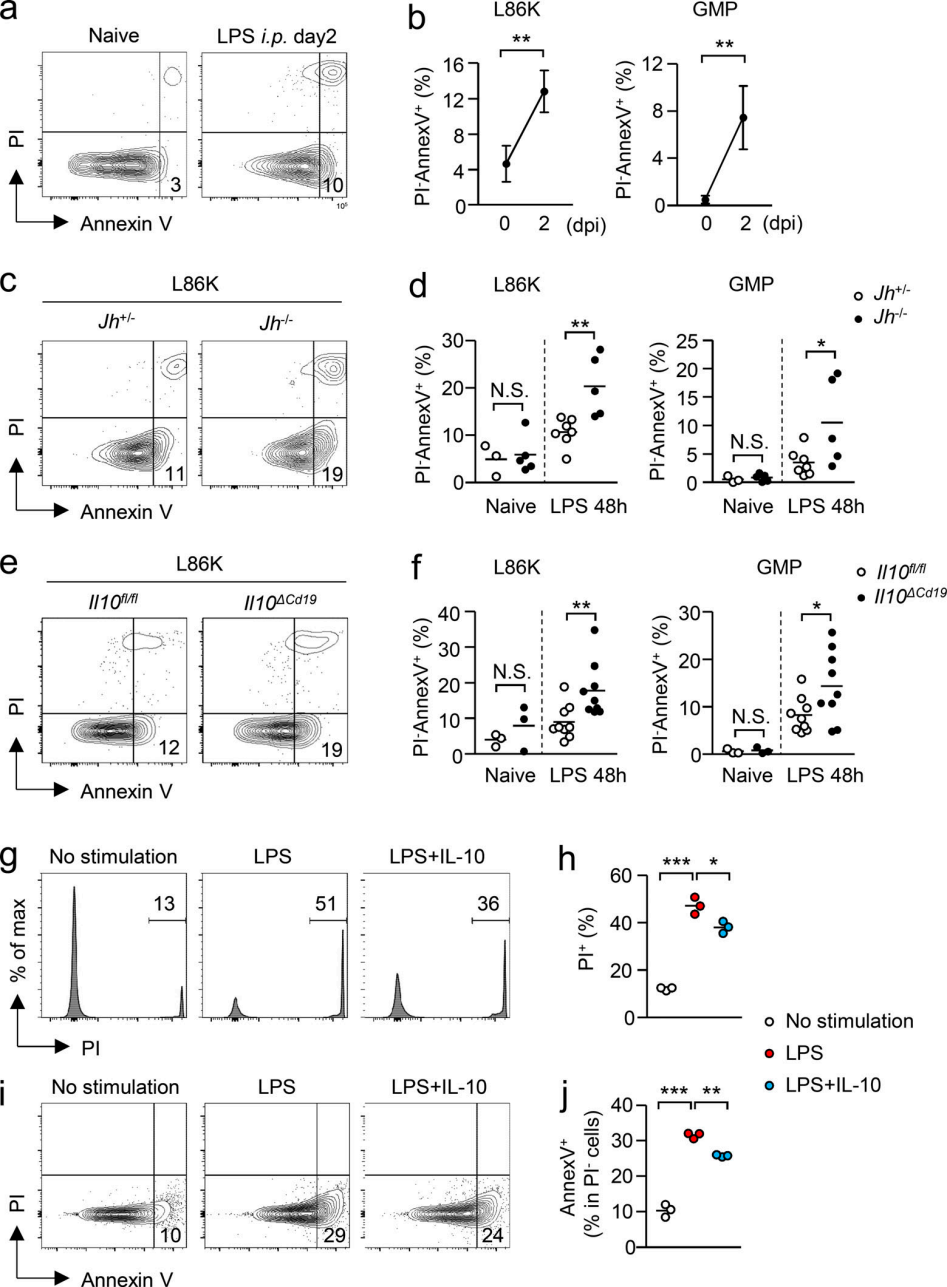
**B cell–derived IL-10 protects hematopoietic progenitors from apoptosis. (a and b)** Apoptotic cell ratios in L86Ks before and after LPS treatment. LPS (5 mg/kg) was intraperitoneally injected into WT C57BL/6 mice and the frequency of early apoptotic (Annexin V^+^ PI^−^) cells in L86Ks and GMPs was examined. Representative FCM plots are shown in panel a. *n* = 3 for the naive group and *n* = 4 for the LPS-treated group. **(c and d)** Frequency of apoptotic cells in L86Ks and GMPs obtained from *Jh*^−/−^ and *Jh*^+/−^ mice before and 2 d after LPS treatment. Representative FCM plots are shown in panel c and the frequency of apoptotic (Annexin V^+^ PI^−^) cells is shown in panel d. *n* = 7 for *Jh*^+/−^ mice and *n* = 5 for *Jh*^−/−^ mice. **(e and f)** Frequency of apoptotic cells in L86Ks and GMPs obtained from *Il10*^*fl/fl*^ mice and from *Il10*^*ΔCd19*^ mice before and 2 d after LPS treatment. Representative FCM plots are shown in panel e and the frequency of apoptotic (Annexin V^+^ PI^−^) cells is shown in panel f. *n* = 9 per group. **(g–j)** Preventing cell death of myeloid progenitors by IL-10. GMPs obtained from the BM of WT mice were stimulated with LPS (1 μg/ml) in the presence or absence of IL-10 (50 ng/ml) for 3 h. The frequency of PI^+^ necrotic cells is shown in panels g and h. Frequencies of Annexin V^+^ apoptotic cells in PI^−^ live cells are shown in panels i and j. Representative FCM plots are shown in panels g and i. *n* = 3 per group. N.S., not significant (P > 0.05), * P < 0.05, ** P < 0.01, *** P < 0.001 (Student’s *t* test [b, d, and f]). Data are representative of two independent experiments (g–j) or from two (a–d) or three (e and f) independent experiments (error bars, SD [b]). Symbols represent individual mice.

To clarify the mechanism by which B cell–derived IL-10 increases hematopoietic progenitors to enhance EM, we examined the expression of inflammatory cytokines and growth factors crucial for EM (Chiba et al., 2018; Marino et al., 1997; Wegenka et al., 2007) in *Il10*^*ΔCd19*^ mice and in *Il10*^*fl/fl*^ mice. However, no significant difference was observed in serum cytokine levels between *Il10*^*ΔCd19*^ and *Il10*^*fl/fl*^ mice (Fig. S4 f). In addition, an IL-10 deficiency in B cells did not alter the serum level of IL-10, suggesting that B cells do not contribute to the IL-10–mediated suppression of systemic inflammation at least early after LPS treatment (Fig. S4 f). IL-10 prevents cell death during infections (Balcewicz-Sablinska et al., 1999; Codo et al., 2018; Gurung et al., 2015; Stacey et al., 2011). As LSKs differentiated into monocytes and neutrophils within 4 d after LPS treatment (Fig. S4, g–i) and since hematopoietic progenitors, including LSKs and GMPs, express IL-10 receptor (IL-10R; Fig. S4 j), we hypothesized that B cell–derived IL-10 prevents the cell death of hematopoietic progenitors prior to the generation of myeloid cells. Thus, we examined the frequency of early apoptotic cells (Annexin V^+^ PI^−^) in L86Ks and GMPs 2 d after LPS treatment. As anticipated, following LPS treatment, the ratios of apoptotic cells increased in both types of progenitors in control mice (Fig. 6, a and b), which were further enhanced in *Jh*^−/−^ mice (Fig. 6, c and d) and in *Il10*^*ΔCd19*^ mice (Fig. 6, e and f). On the other hand, the apoptotic cell ratios of neutrophils and monocytes were unaltered between *Il10*^*ΔCd19*^ and *Il10*^*fl/fl*^ mice 2 d after LPS treatment (Fig. S4 k). To confirm whether IL-10 can directly prevent the cell death of myeloid progenitors, we stimulated GMPs with LPS in the presence or absence of IL-10 for 3 h. As expected, LPS stimulation significantly increased the frequency of PI^+^ dead cells and Annexin V^+^ apoptotic cells (Fig. 6, g–j), which was partially but significantly attenuated by adding IL-10 (Fig. 6, g–j). Thus, B cell–derived IL-10 protects hematopoietic progenitors from collateral damage and contributes to the effective induction of EM.

### IL-10 biases hematopoiesis to myelopoiesis

**Figure 7.**
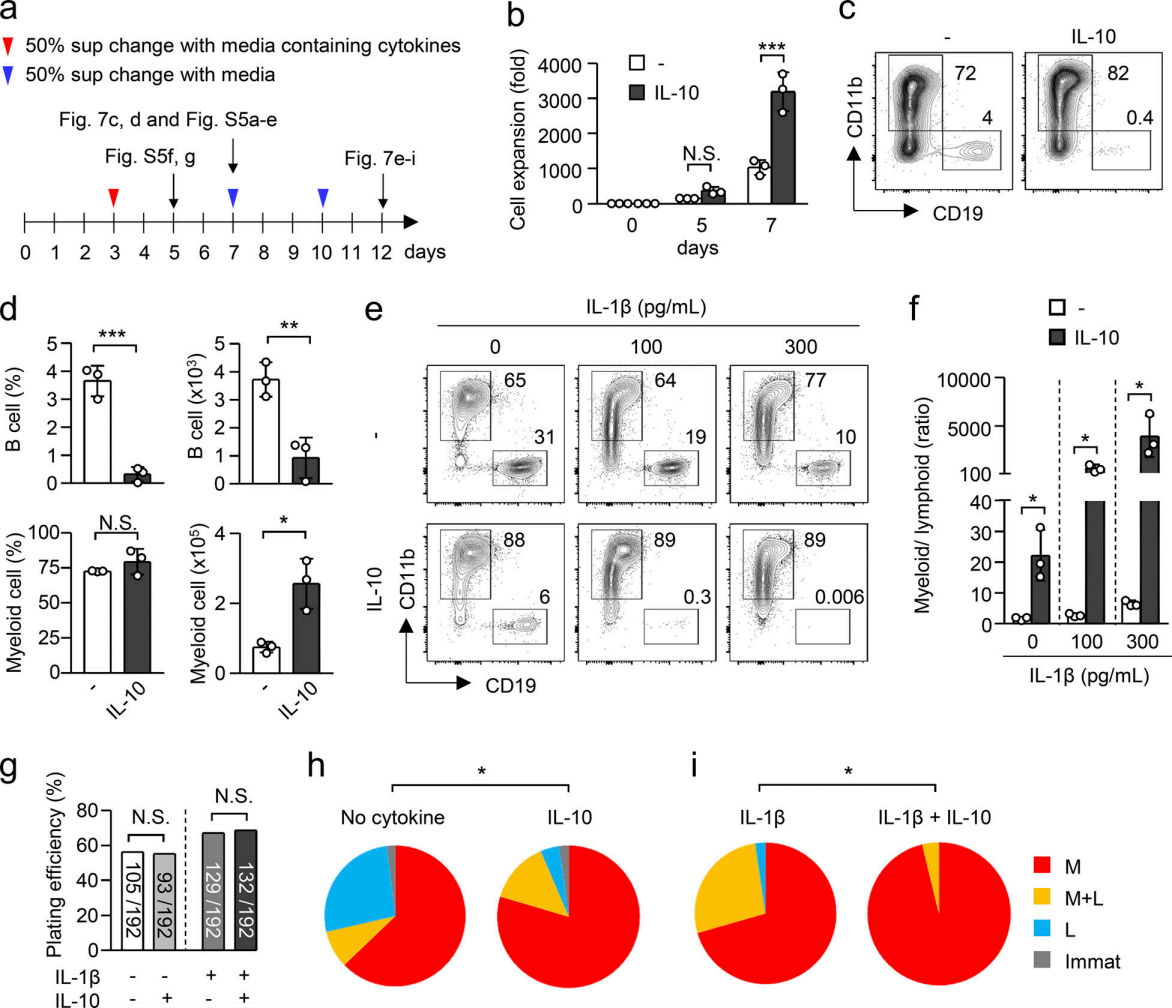
**IL-10 induces myeloid-biased hematopoiesis. (a)** Schedule for the culture of LSKs with cytokines. LSKs obtained from the BM of naive C57BL/6 mice were cultured on MS-5 stromal cells in the presence or absence of cytokines for 5, 7, or 12 d. **(b)** Fold change of cell number of cultured LSK-derived cells. LSK cells obtained from the BM of naive C57BL/6 mice were cultured with or without IL-10 (10 ng/ml) for 5 or 7 d, and the numbers of cells were counted by FCM with cell-counting beads. *n* = 3 per group. **(c and d)** Enhancement of myeloid-biased hematopoiesis by IL-10. LSKs were cultured on MS-5 cells in the presence or absence of IL-10 (10 ng/ml) for 7 d. The numbers (right panels) and frequencies (left panels) of B cells (CD11b^−^CD19^+^; upper panels) and myeloid cells (CD11b^+^CD19^−^; lower panels) are shown in panel d. Representative FCM plots are shown in panel c. *n* = 3 per group. **(e and f)** Cooperative effect of IL-10 and IL-1β to induce myeloid-biased hematopoiesis. LSKs obtained from the BM of naive C57BL/6 mice were cultured on MS-5 cells in the presence or absence of IL-10 (10 ng/ml) and/or IL-1β (100 or 300 pg/ml) for 12 d. The frequencies of B cells (CD11b^−^CD19^+^; upper panels) and myeloid cells (CD11b^+^CD19^−^; lower panels) are shown in panel f. Representative FCM plots are shown in panel e. *n* = 3 per group. **(g–i)** Single-cell culture of LSKs with IL-10. LSKs isolated from the BM of naive C57BL/6 mice were singly plated on MS-5 cells and cultured in the presence or absence of IL-10 (10 ng/ml) and/or IL-1β (100 pg/ml) for 12 d. Plating efficiencies are shown in panel g and frequencies of colonies for myeloid (CD11b^+^CD19^−^; denoted as M), lymphoid (CD11b^−^CD19^+^; denoted as L), and immature cells (CD11b^−^CD19^−^; denoted as Immat) are shown in panels h and i. N.S., not significant (P > 0.05), * P < 0.05, ** P < 0.01, *** P < 0.001 (Student’s t test [d], χ2 test [g–i], or one-way ANOVA [b and f]). Data are representative of two independent experiments (b–f) or from two independent experiments (g–i); (error bars, SD [b, d, and f]). Symbols represent individual mice.

**Figure S5.**
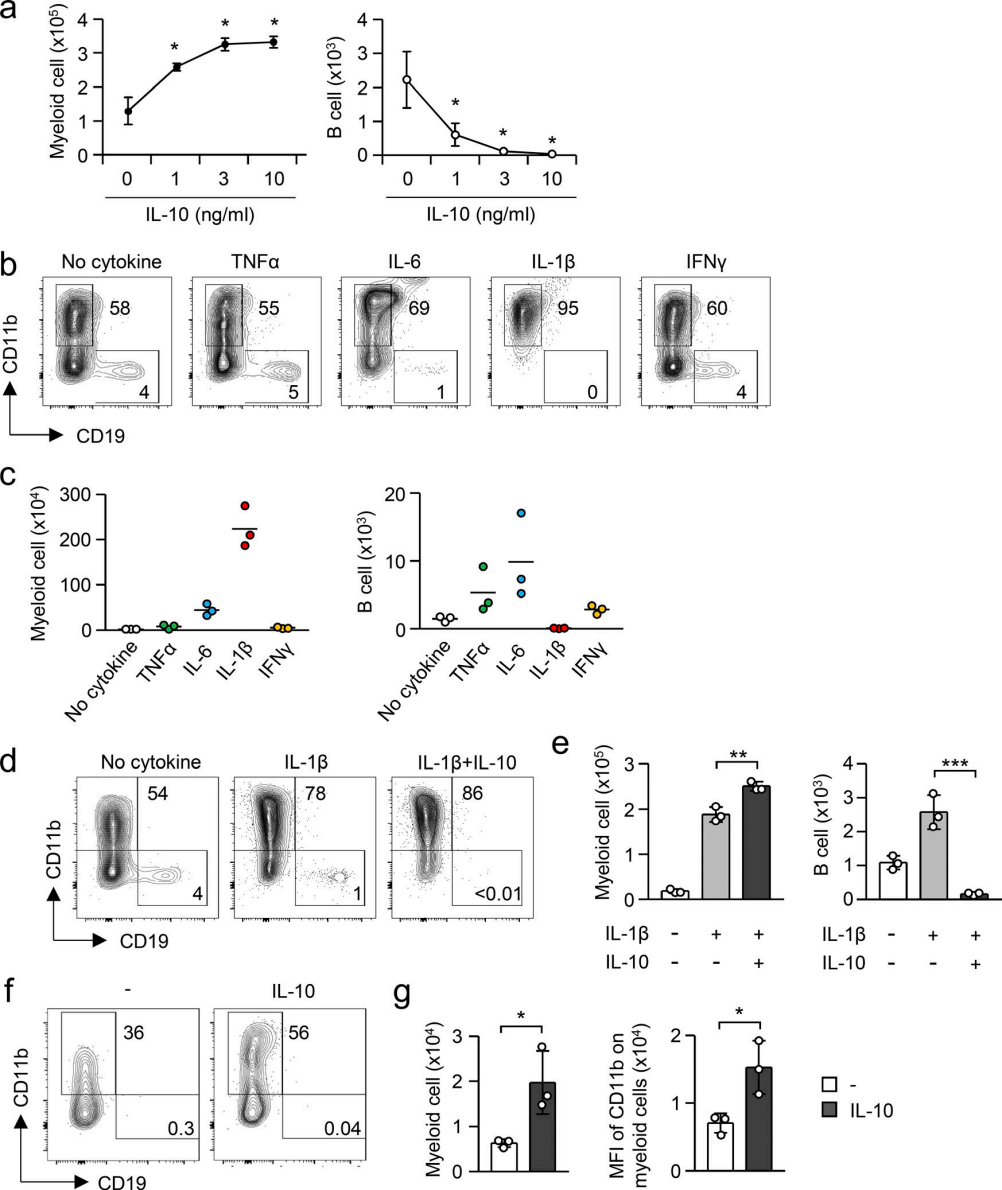
**IL-10 enhances myeloid cell differentiation. (a)** Induction of myeloid-biased hematopoiesis by IL-10. LSKs were cultured with MS-5 stromal cells in the presence of the indicated concentration of IL-10 for 7 d. Numbers of myeloid cells (CD11b^+^CD19^−^) and B cells (CD11b^−^CD19^+^) were examined by flow cytometry. *n* = 3 per group. **(b and c)** Induction of myeloid-biased hematopoiesis by inflammatory cytokines. LSKs were cultured with Ms-5 cells in the presence of IL-10 (10 ng/ml) for 7 d. Frequencies and numbers of myeloid cells and B cells were examined by flow cytometry. *n* = 3 per group. **(d and e)** Enhanced myelopoiesis by IL-1β and IL-10. LSKs were cultured with Ms-5 cells in the presence or absence of IL-1β (50 pg/ml) and IL-10 (10 ng/ml) for 7 d. Numbers of myeloid cells and B cells were examined by flow cytometry. *n* = 3 per group. Representative FCM plots are shown in panel d. **(f and g)** Accelerated differentiation of myeloid cells by IL-10. LSKs were cultured with Ms-5 cells in the presence or absence of IL-10 (10 ng/ml) for 5 d as shown in panel a. Numbers of myeloid cells and mean fluorescence intensity (MFI) of CD11b in myeloid cells were examined by flow cytometry. *n* = 3 per group. Representative FCM plots are shown in panel f. * P < 0.05, ** P < 0.01, *** P < 0.001 (Student’s *t* test [g] or one-way ANOVA [a and e]). Data are representative of two independent experiments (error bars, SD [a, e, and g]). Symbols represent individual mice.

We then tested the efficacy of IL-10 in the cell fate of hematopoietic progenitors in LPS-free conditions, which can exclude the impact of IL-10 in cell death. We cultured LSKs with MS-5 stromal cells in the presence or absence of IL-10 for 5, 7, or 12 d (Fig. 7 a). The addition of IL-10 significantly enhanced cell expansion (Fig. 7 b). At day 7 after starting the culture, the frequency and number of CD19^+^ B lymphocytes were significantly reduced and the number of CD11b^+^ myeloid cells was conversely increased (Fig. 7, c and d, and Fig. S5 a), suggesting that IL-10 can induce myeloid-biased hematopoiesis. We further asked whether IL-10 promotes myelopoiesis even in the presence of inflammatory cytokines that cause EM. Because IL-1β is a representative cytokine that drives EM (Pietras et al., 2016) and showed the most remarkable activity to induce myeloid-biased hematopoiesis, at least in this culture setting (Fig. S5, b and c), we cultured LSKs in the presence of IL-10 and/or IL-1β. The addition of IL-1β significantly decreased the frequency of B cells and increased the frequency of myeloid cells at days 7 and 12 (Fig. 7 e and Fig. S5 d). Importantly, the effect of IL-1β to induce myeloid-biased hematopoiesis was further amplified by IL-10 (Fig. 7, e and f; and Fig. S5, d and e). Thus, IL-10 can promote myeloid-biased hematopoiesis.

Our results suggested that IL-10 protects hematopoietic progenitors from cell death (Fig. 6). Therefore, we evaluated whether IL-10 induces myeloid-biased hematopoiesis by selectively promoting the survival of myeloid-committed progenitors or whether IL-10 directs hematopoietic progenitors to myeloid cell differentiation. At day 5 of LSK culture, we found that IL-10 increased not only the number of CD11b^+^ cells but also the mean fluorescence intensity of CD11b (Fig. S5, f and g), indicating the acceleration of myeloid cell differentiation. Importantly, single cell-culture analysis of LSKs revealed that IL-10 did not alter the plating efficiency of LSKs in the absence or presence of IL-1β (Fig. 7 g), which suggests that IL-10 has no impact on the survival of LSKs seeded in culture without harmful stimulation. However, IL-10 significantly increased the frequency of myeloid-related colonies (shown as red and orange areas) regardless of IL-1β (Fig. 7, h and i). Taken together, in addition to the antiapoptotic function in harmful conditions, we conclude that IL-10 alters the cell fate of hematopoietic progenitors to effectively induce EM.

### Direct action of IL-10 on hematopoietic progenitors to enhance EM

**Figure 8.**
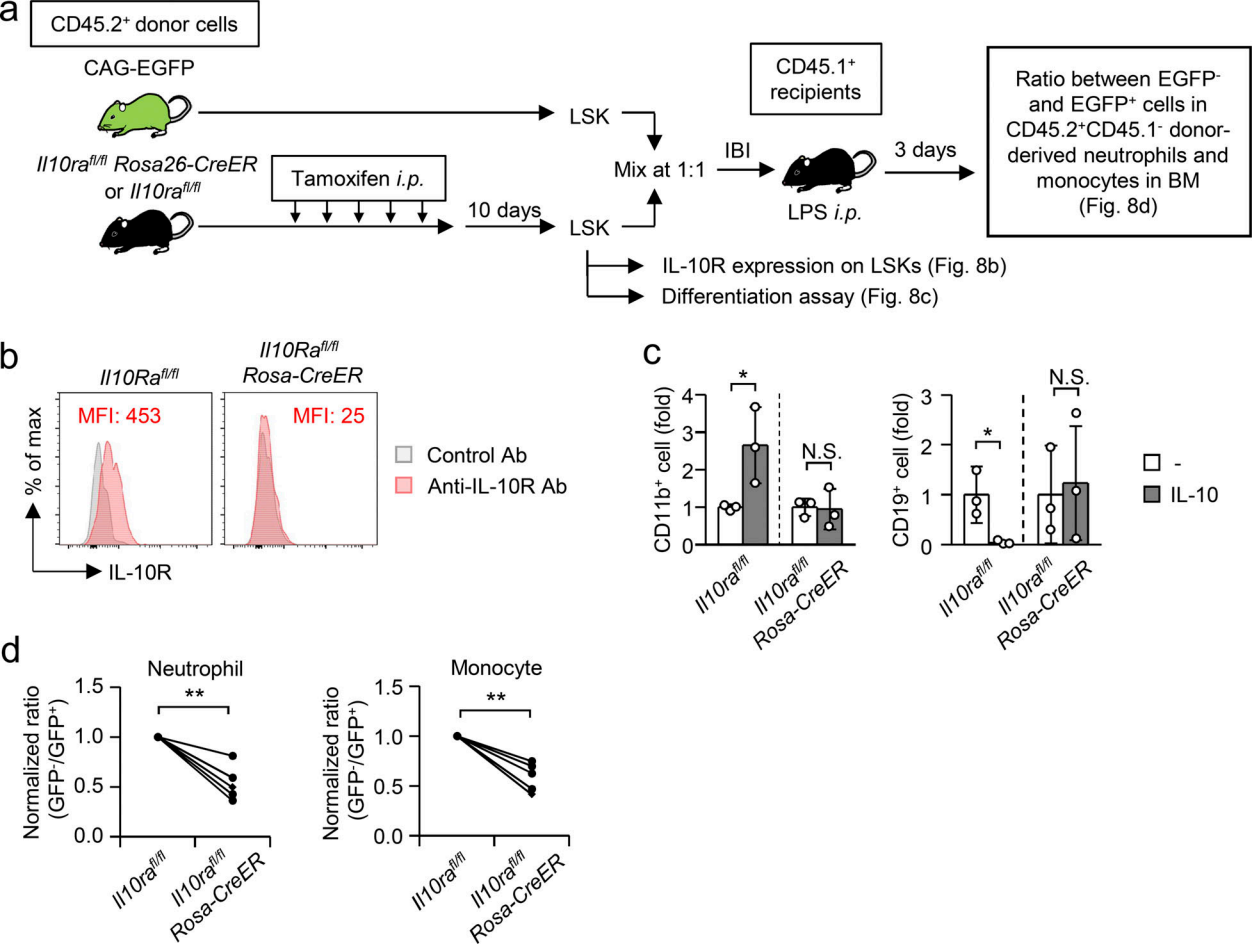
**IL-10 directly stimulates hematopoietic progenitors to enhance EM. (a)** Experimental strategy to examine the impact of IL-10R deficiency in LSKs on EM induction. **(b)** Deletion of the IL-10R on LSKs by tamoxifen treatment. Tamoxifen (2 mg) was injected into *Il10ra*^*flox/flox*^*; Rosa26-CreER* or *Il10ra*^*flox/flox*^ mice daily for 5 d and the expression of IL-10R was confirmed 10 d after last tamoxifen treatment using FCM. Data are representative of two independent experiments. MFI, mean fluorescence intensity. **(c)** Lack of IL-10R on LSKs canceled the efficacy of IL-10 to induce myeloid-biased hematopoiesis. 10 d after tamoxifen treatment of *Il10ra*^*flox/flox*^*; Rosa26-CreER* or *Il10ra*^*flox/flox*^ mice, LSKs were isolated and cultured with MS-5 stromal cells in the presence or absence of recombinant IL-10 (rIL-10, 10 ng/ml) for 7 d to examine the generation of CD11b^+^ myeloid cells and CD19^+^ B lymphocytes. *n* = 3 each group. **(d)** LSKs obtained from tamoxifen-treated *Il10ra*^*flox/flox*^*; Rosa26-CreER* mice or *Il10ra*^*flox/flox*^ mice were mixed at a 1:1 ratio with LSKs obtained from naive CAG-EGFP mice and were directly transplanted into the BM of CD45.1-expressing recipient mice. 3 d after LPS treatment of the recipient mice, the ratio between EGFP^−^ and EGFP^+^ cells in donor-derived neutrophils (CD11b^+^Ly6C^+^ Ly6G^+^) and monocytes (CD11b^+^Ly6C^hi^ Ly6G^−^) in the BM was determined. *n* = 5 each group. N.S., not significant (P > 0.05), * P < 0.05, ** P < 0.01 (Student’s *t* test). Data are representative of two independent experiments (b and c) or from three independent experiments (d) (error bars, SD [b]). Symbols represent individual mice.

To directly evaluate the importance of IL-10R–mediated signaling in hematopoietic progenitors for EM induction, we next prepared tamoxifen-inducible *Il10ra*-deficient mice (*Il10ra*^*fl/fl*^*Rosa26-CreER* mice). 10 d after tamoxifen treatment (Fig. 8 a), the expression of IL-10R on LSKs was almost completely lost in *Il10ra*^*fl/fl*^*Rosa26-CreER* mice (Fig. 8 b). When the LSKs were cultured with IL-10, the efficacy of IL-10 to induce myeloid-biased hematopoiesis was completely canceled (Fig. 8 c), suggesting that IL-10 directly acts on hematopoietic progenitors to skew their cell fate to myeloid cell differentiation ex vivo. To demonstrate the direct action of IL-10 on hematopoietic progenitors in EM in vivo, LSKs from tamoxifen-treated *Il10ra*^*fl/fl*^*Rosa26-CreERT* mice or their littermate control (*Il10ra*^*fl/fl*^) mice were mixed with LSKs from CAG-EGFP mice at a 1:1 ratio and were then directly injected into the BM of recipient CD45.1^+^ B6.SJL mice (Fig. 8 a). 3 d after LPS injection, the ratios between EGFP^−^ and EGFP^+^ cells in the donor-derived neutrophils and monocytes were compared. Of note, the ratios of EGFP^−^ cells in donor-derived neutrophils and monocytes were significantly reduced when *Il10ra*-deficient LSKs were used as donor cells (Fig. 8 d), demonstrating that direct IL-10R signaling in hematopoietic progenitors boosts EM in vivo.

### B cell–derived IL-10 boosts EM to enhance the clearance of invading microbes

**Figure 9.**
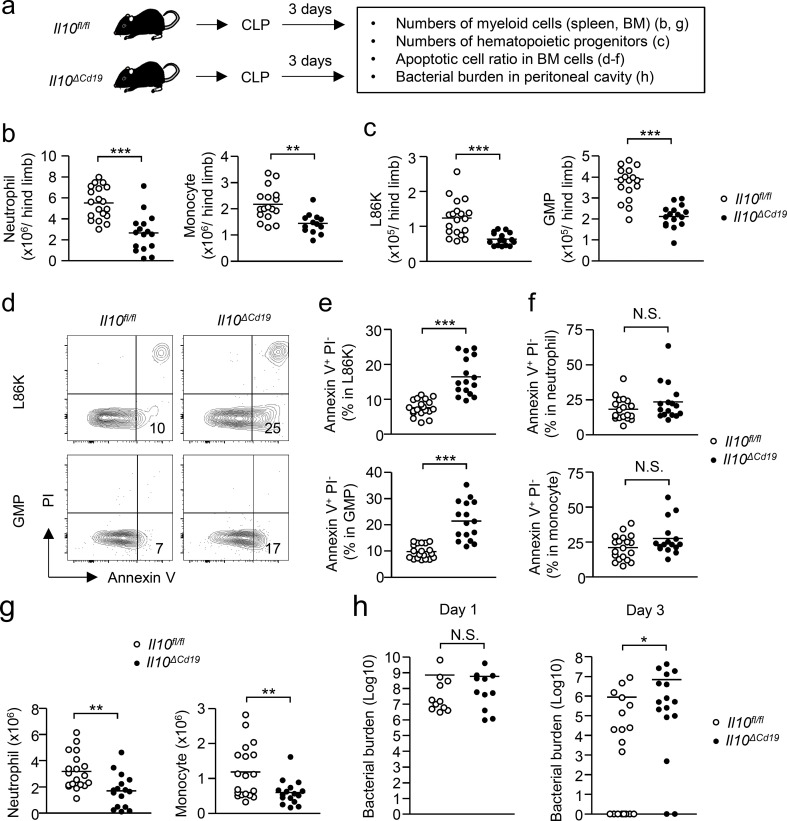
**B cell–derived IL-10 enhances EM and boosts anti-microbial immune responses. (a)** Experimental strategy of CLP. CLP was induced in *Il10*^*ΔCd19*^ mice and in their littermate control (*Il10*^*fl/fl*^) mice, which were sacrificed and analyzed at day 3. **(b)** Influence of IL-10 derived from B cells in myeloid cell generation induced by CLP. Number of neutrophils (CD11b^+^Ly6C^+^ Ly6G^+^; left panel) and monocytes (CD11b^+^Ly6C^hi^ Ly6G^−^; right panel) in the BM of *Il10*^*fl/fl*^ and *Il10*^*ΔCd19*^ mice were examined 3 d after CLP induction. *n* = 19 for *Il10*^*fl/fl*^ mice and *n* = 16 for *Il10*^*ΔCd19*^ mice. **(c)** Influence of B cell–derived IL-10 on the number of hematopoietic progenitors. Numbers of L86Ks and GMPs in *Il10*^*fl/fl*^ and *Il10*^*ΔCd19*^ mice were examined 3 d after CLP induction. *n* = 19 for *Il10*^*fl/fl*^ mice and *n* = 16 for *Il10*^*ΔCd19*^ mice. **(d–f)** Influence of B cell–derived IL-10 in the survival of hematopoietic progenitors. Frequencies of early apoptotic cells (Annexin V^+^ PI^−^) were examined in L86Ks and GMPs (d and e) and neutrophils and monocytes (f) in the BM of *Il10*^*fl/fl*^ mice and *Il10*^*ΔCd19*^ mice 3 d after CLP induction. Representative FCM plots of GMPs and L86K cells are shown in panel d. *n* = 19 for *Il10*^*fl/fl*^ mice and *n* = 16 for *Il10*^*ΔCd19*^ mice. **(g)** Influence of IL-10 derived from B cells in the recruitment of innate immune cells in the periphery. Numbers of neutrophils (left panel) and monocytes (right panel) in the spleens of *Il10*^*fl/fl*^ and *Il10*^*ΔCd19*^ mice were examined 3 d after CLP induction. *n* = 19 for *Il10*^*fl/fl*^ mice and *n* = 16 for *Il10*^*ΔCd19*^ mice. **(h)** Increased clearance of microbiota by IL-10 produced by B cells. Bacterial burdens in the peritoneal cavity of *Il10*^*fl/fl*^ mice and *Il10*^*ΔCd19*^ mice were examined 1 and 3 d after CLP induction. Day 1: *n* = 11 for *Il10*^*fl/fl*^ mice and *Il10*^*ΔCd19*^ mice, Day 3: *n* = 19 for *Il10*^*fl/fl*^ mice and *n* = 16 for *Il10*^*ΔCd19*^ mice. N.S., not significant (P > 0.05), * P < 0.05, ** P < 0.01, *** P < 0.001 (Student’s *t* test). Data are from eight (b–g, right panel in h) or five (left panel in h) independent experiments. Symbols represent individual mice.

Finally, to determine whether the EM enhanced by B cell–derived IL-10 contributes to host protection during infections, we induced CLP, the most frequently used model for human sepsis, in *Il10*^*ΔCd19*^ mice and their littermate control (*Il10*^*fl/fl*^) mice (Fig. 9 a). Compared with CLP-induced *Il10*^*fl/fl*^ mice, CLP-induced *Il10*^*ΔCd19*^ mice showed an impaired generation of neutrophils, monocytes, and their progenitors, L86Ks and GMPs (Fig. 9, b and c), with higher proportions of apoptotic cells in L86Ks and GMPs (Fig. 9, d and e). As observed in LPS-induced EM, the frequency of apoptotic cells in neutrophils and monocytes was comparable between *Il10*^*fl/fl*^ mice and *Il10*^*ΔCd19*^ mice after CLP induction (Fig. 9 f). The impaired EM in *Il10*^*ΔCd19*^ mice led to a significant decrease of neutrophils and monocytes in the periphery (Fig. 9 g), resulting in a higher bacterial burden at day 3, but not at day 1 after CLP induction (Fig. 9 h). These results suggest that B cell–derived IL-10 enhances the clearance of microbes by reinforcing myelopoiesis.

**Figure 10.**
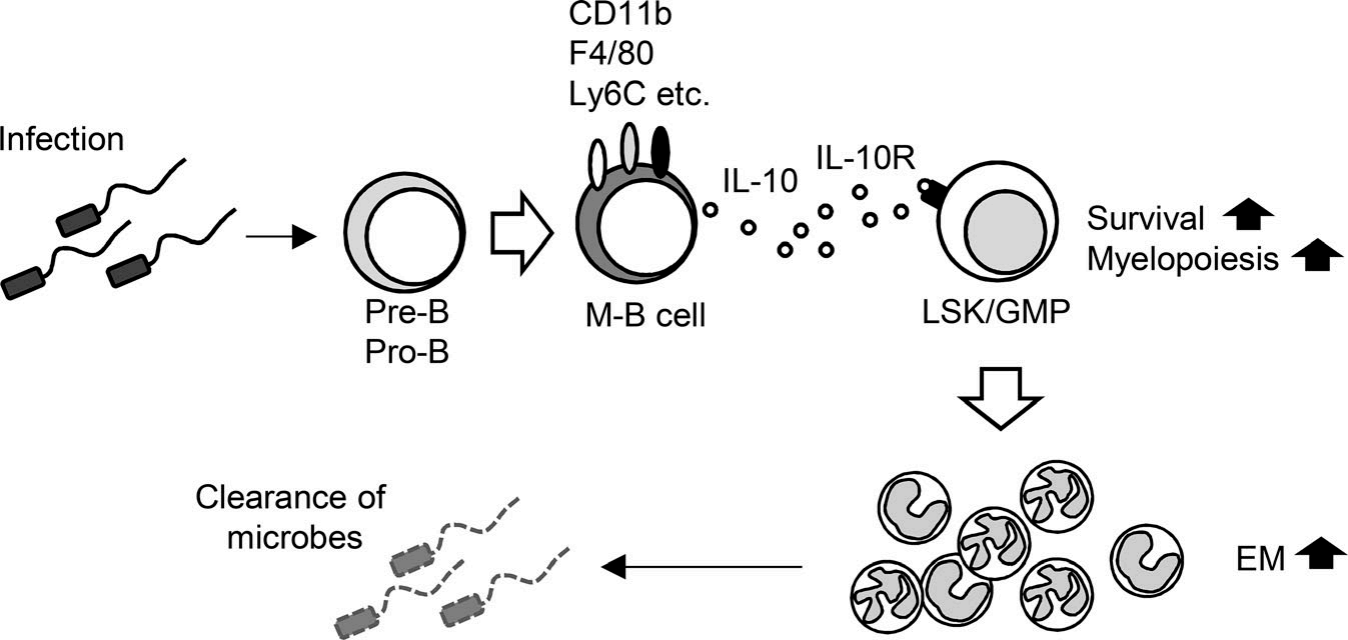
Schematic illustration of enhanced host protection through IL-10 produced by M-B cells.

Taken together, the results show that M-B cells develop quickly from B cell precursors as a producer of IL-10, which boosts EM by directly promoting the survival and myeloid-biased differentiation of hematopoietic progenitors, leading to the effective elimination of invasive pathogens (Fig. 10).

## Discussion

Innate immunity is an ancient system that is observed in a wide variety of animals including invertebrates. The supply of innate immune cells, such as neutrophils and monocytes, is controlled by hematopoiesis. Early after systemic infections, large amounts of myeloid cells are generated by EM, which provides a quick supply of innate immune cells to the infected sites. In contrast, lymphocytes are numerically and functionally maintained in other lymphoid organs, such as the thymus, spleen, and lymph nodes, rather than in the BM. As the number of lymphocytes is dramatically decreased in the BM during EM, the impact of lymphocytes on the induction of EM is considered to be minimal.

IL-10 is an anti-inflammatory molecule that maintains tissue homeostasis. Tregs and plasma cells have been reported to be sources of IL-10 in the BM (Fujisaki et al., 2011; Meng et al., 2019). However, it had not been clear which cells supply IL-10 in the BM after infection. We found that Tregs and plasma cells disappeared quickly in the BM of LPS-treated mice. Instead, M-B cells that are phenotypically distinguishable from other B cell subsets quickly emerged as a major producer of IL-10 in the BM during EM. M-B cells show the morphological features of lymphocytes and could be generated from B cell precursors in the BM. In addition, M-B cells are not found in *Rag2*^*−/−*^ mice, clearly suggesting that M-B cells are of lymphoid origin. Regulatory B cells are a defined population based on their immune regulatory functions, including the production of IL-10 (Rosser and Mauri, 2015). Thus, it may be reasonable to regard M-B cells as a type of regulatory B cell. Our results show that LPS treatment increases CD11b expression even in pro- and pre-B cells, which do not express mature B cell receptor (BCR). Thus, the emergence of M-B cells may not depend on BCR-mediated stimulation. Supporting this notion, LPS is a polyclonal B cell activator (Dziarski, 1982; Montes et al., 2007). Thus, it is likely that the BCR repertoire of M-B cells is polyclonal and is similar to that of CD11b^−^ B cells.

Although IL-10 deficiency in B cells inhibited EM after LPS injection or CLP induction, it did not affect the serum levels of inflammatory cytokines after LPS treatment. This result is consistent with a previous report showing that a B cell–specific deletion of IL-10 does not change the mortality of LPS-induced sepsis, although a total lack of IL-10 increased the severity of sepsis (Berg et al., 1995; Madan et al., 2009). In addition, Cardoso et al. (2021) reported that, in infection-free mice, IL-10 that is overexpressed in stromal cells indirectly stimulates T cells to produce IFN-γ, which acts on hematopoietic progenitor cells to induce myelopoiesis. However, the pathophysiological roles of IL-10 in infection-induced EM remain to be elucidated. In this context, using infection-induced EM models, we suggest that M-B cell–derived IL-10 directly acts on hematopoietic progenitors to enhance EM by two different mechanisms: (1) by decreasing the cell death of hematopoietic progenitors and (2) by promoting myeloid-biased hematopoiesis. Although an antiapoptotic role of IL-10 was previously reported in various types of cells, including hematopoietic progenitors (Dhingra et al., 2011; Moore et al., 2001; Weber-Nordt et al., 1996; Zhou et al., 2001), the efficacy of IL-10 to trigger myeloid-biased hematopoiesis was unexpected because EM is known to be induced by proinflammatory factors rather than by anti-inflammatory factors such as IL-10. The suppression of cell death by IL-10 seems to be cell type dependent. B cell–derived IL-10 did not affect the apoptosis of neutrophils or monocytes, whereas it significantly decreased the apoptosis of hematopoietic progenitors. Our results also indicate that signaling through the IL-10R directly enhances the survival of hematopoietic progenitors and their myeloid-biased differentiation.

A lack of B cell–derived IL-10 significantly prevented the efficient supply of innate immune myeloid cells and impaired the clearance of bacteria in CLP-induced mice. As M-B cells were rarely observed in the BM of naive mice, the mechanism that promotes myelopoiesis may only be driven during pathogenic conditions such as acute infections. Indeed, the lack of B cells or B cell–derived IL-10 did not influence the number of myeloid cells in naive conditions.

The emergence of the adaptive immune system in vertebrates has led to coordination with innate immunity, allowing the induction of specific defensive immunity against pathogens. In addition to the well-known interactions between the innate and adaptive immune systems, our findings suggest the presence of a unique interplay by which the adaptive immune cells promote the on-demand innate immune myeloid cell production from hematopoietic progenitors and effectively counter infectious environments until the adaptive immune system is ready. Our findings highlight the crucial role of a new B lymphocyte subset in boosting EM and further imply the evolutionary reinforcement of EM by adaptive immune cells, which is probably important for vertebrates who live on land with dramatically increased opportunities for contact with pathogens.

## Materials and methods

### Mice

C57BL/6J (B6) mice were obtained from Japan Slc Inc. (Hamamatsu); B6.SJL-ptprca (B6.SJL, Strain #: 4007) mice congenic at the CD45 locus (CD45.1^+^CD45.2^−^) and *Jh*^−/−^ mice (Strain #: 17758) were from Taconic. *Il10ra* ^*fl/fl*^ (Strain #: 028146; Liu et al., 2012), CAG-EGFP (Strain #: 003291), *Cd19-Cre* (Strain #: 006785), *Tlr4*^*flox/flox*^ (Strain #: 024872), and *Rosa26-CreER* (Strain #: 008463) mice were from Jackson Laboratories. *Il-10 Venus* and *Il10*^*fl/fl*^ mice were used as previously reported (Atarashi et al., 2011; Roers et al., 2004). All mice were maintained in our specific pathogen–free animal facility, and all experiments using mice were approved by the Institutional Animal Care Committee of the Tokyo Medical and Dental University.

### Real-time PCR and ELISA analysis

Total mRNAs were reverse-transcribed to cDNAs and gene expression levels were determined using a Light Cycler 480 and SYBR Green I Master (04707516001; Roche Diagnostics). The values were normalized by the expression of β-actin. Specific primers used for real-time PCR are as follows: *Il10* forward: 5′-GGT​TGC​CAA​GCC​TTA​TCG​GA-3′, *Il10* reverse: 5′-ACC​TGC​TCC​ACT​GCC​TTG​CT-3′, *Tbx21* forward: 5′-AAT​CGA​CAA​CAA​CCC​CTT​TG-3′, *Tbx21* reverse: 5′-AAC​TGT​GTT​CCC​GAG​GTG​TC-3′, *Gata2* forward: 5′-CAA​GAA​AGG​GGC​TGA​ATG​TTT​CG-3′, *Gata2* reverse: 5′-GTG​TCC​CAC​AGG​TGC​CAT​G-3′, *Actb* forward: 5′-TGT​TAC​CAA​CTG​GGA​CGA​CA-3′, *Actb* reverse: 5′-CTG​GGT​CAT​CTT​TTC​ACG​GT-3′. To evaluate the secretion of IL-10 from BM B cells, B cell populations or total BM cells were obtained from WT or *Cd19-Cre Il10*^*fl/fl*^ mice before or 24 or 48 h after LPS treatment (L2880-100MG, 5 mg/kg; Sigma-Aldrich) and were cultured in RPMI-1640 media containing 10% FBS in the presence of LPS (100 ng/ml) for 24 h. IL-10 concentrations in the supernatants were determined using an IL-10 ELISA kit (Biolegend). Levels of TNF-α, IL-6, IL-1β, G-CSF, and GM-CSF in the serum 2 h after LPS injection (5 mg/kg) were measured using ELISA kits purchased from R&D Systems.

### Cytological analysis

CD19^+^B220^hi^IgM^+^ mature B cells were obtained from the BM of naive mice, and CD11b^−^CD19^+^B220^hi^IgM^+^ and CD11b^−^CD19^+^B220^hi^IgM^+^ cells were obtained from the BM 48 h after LPS treatment. Cells were stained with Diff Quik (Sysmex) and the diameters of nuclei in each population were evaluated by ImageJ.

### Systemic infection induced by the CLP model

Mice were anesthetized by intraperitoneal injection of an anesthetic mixture of medetomidine, midazolam, and butorphanol. The abdomen of each mouse was shaved and a laparotomy was performed. The cecum was exposed and tightly ligated 0.5 cm from the distal end. The ligated cecum was then perforated once with a 19 G needle. The cecum was returned to the peritoneal cavity after gentle squeezing to extrude a small amount of feces from the perforated sites. The peritoneum was sutured and the skin was closed using a clip. 3 d after CLP induction, the mice were sacrificed and the frequencies of neutrophils and monocytes in the BM and the peritoneal cavity and the peritoneal bacterial burden were examined.

### Evaluation of apoptosis in myeloid cells and progenitors

BM cells were obtained from mice 2 d after the intraperitoneal injection of LPS (5 mg/kg). After staining cell surface markers for myeloid or LSK cells, cells were stained with a PE-conjugated anti-Annexin V antibody (Thermo Fisher Scientific) and propidium iodide (PI) and were analyzed by flow cytometry (FCM). For culture experiments, GMPs obtained from naive WT mice were stimulated with LPS (1 μg/ml) in the presence or absence of IL-10 (20 ng/ml) for 3 h. The cells were then stained with an anti-Annexin V antibody and PI and were analyzed by FCM.

### Culture of LSKs with MS-5 stromal cells

LSKs isolated from naive WT mice, tamoxifen-treated *Il10ra*^*fl/fl*^ mice, or *Il10ra*^*fl/fl*^*:Rosa26-CreERT* mice were cultured on a layer of MS-5 stromal cells in RPMI-1640 media containing 20% FBS in the presence or absence of IL-10 (10 ng/ml), IL-1β (0.05–10 ng/ml), IL-6 (10 ng/ml), IFN-γ (10 ng/ml), and/or TNFα (10 ng/ml) for 5–12 d. For single-cell cultures of LSKs, LSKs were singly sorted into 96-well plates coated with MS-5 stromal cells and were cultured for 12 d. Half of each supernatant was exchanged with fresh media containing cytokines at day 3 and was changed with cytokine-free fresh media at days 7 and 10, as shown in Fig. 7 a. The frequencies and numbers of myeloid cells and B cells were examined using FCM. For pretreatment of LSKs with IL-10, LSKs obtained from naive WT mice were cultured in the presence of IL-10 (10 ng/ml) for 15 h. After the culture, live cells were isolated and seeded on MS-5 cell–coated plates and cultured for 7 d.

### FCM and cell sorting

BM, spleens, and peritoneal cells were obtained from mice. After staining with specific antibodies, cells were analyzed using a FACS Aria III or a FACS Canto II (BD Bioscience) and then by FlowJo software (Treestar Inc.). For sorting of B cell lineages, BM cells were stained with PE/Cy5-conjugated antibodies against CD3, CD4, CD8, Gr-1, NK1.1, and TER119. After washing, cells were incubated with anti-Cy5 microbeads (Miltenyi Biotec) and B cells were isolated by negative isolation with Auto MACS (Miltenyi Biotec). BM B cells were then stained with specific antibodies, and specific cell populations were isolated using a FACS Aria III (BD Bioscience). For counting cell numbers in the BM and spleen, CountBrightTM absolute counting beads (Invitrogen) were used. Apoptotic cells were detected using an Annexin V Apoptosis Detection Kit PE (Thermo Fisher Scientific). For RNA-sequencing analysis, CD11b^+^F4/80^+^ peritoneal macrophages were sorted from the peritoneal cavity of naive mice and CD11b^−^ B cells and M-B cells were sorted from the BM 48 h after LPS treatment.

### Intra-BM transplantation

Donor cells were isolated from the BM of CAG-EGFP mice or WT B6 mice. Recipient SJL mice were anesthetized using an anesthetic mixture of medetomidine, midazolam, and butorphanol, after which the donor cells were transplanted into the tibias of the recipient mice. After the intra-BM transplantation, mice were quickly recovered by an injection of atipamezole. In some experiments, mice were treated with LPS (5 mg/kg) 1 or 12 h after the transplantation. For transplantation of LSKs lacking IL-10R, tamoxifen (2 mg/mouse) was intraperitoneally injected into *Il10ra*^*fl/fl*^ or *Il10ra*^*fl/fl*^*:Rosa26-CreERT* mice daily for 5 d. 10 d after the last tamoxifen treatment, the deletion of IL-10R on LSKs was confirmed by FCM. The LSKs were mixed with LSKs obtained from CAG-EGFP mice at a 1:1 ratio and injected into the BM of the tibias. 3 d after the intraperitoneal injection of LPS (5 mg/kg), the ratios between EGFP^−^ and EGFP^+^ cells in donor-derived neutrophils and monocytes were determined in the BM. In another experiment, CD19^+^B220^low^IgM^−^ pre-/pro-B cells obtained from *Tlr4*^*flox/flox*^*; Vav1-cre* and their littermate control (*Tlr4*^*flox/flox*^) mice were transplanted into the BM of each tibia and LPS (5 mg/kg) was injected 12 h after the transplantation. 48 h after LPS treatment, CD11b expression by the donor cells was examined by FCM.

### G-CSF–derived myelopoiesis

Recombinant mouse G-CSF (5 μg/mouse; Biolegend) was intraperitoneally administered to *Il10*^*flox/flox*^*; Cd19-Cre* mice and their littermate control (*Il10*^*flox/flox*^) mice daily for 7 d. 24 h after the last injection, the numbers of neutrophils, monocytes, L86Ks, and GMPs in the spleen and/or BM were examined by FCM.

### Local blockade of IL-10

An anti-mouse IL-10 rat antibody (clone: JES5-2A5) or a control rat IgG1 (1 μg/tibia) was injected into the BM of the left and right tibia, respectively. 4 d after treatment with LPS (5 mg/kg), the numbers of neutrophils and monocytes in the BM of the tibia were determined by FCM.

### Transcriptome library preparation and mRNA sequencing

RNA sequence library preparation, sequencing, mapping, gene expression, and gene ontology (GO) enrichment analyses were performed by DNAFORM (Yokohama). The quality of each total RNA was assessed using a Bioanalyzer (Agilent) to ensure that the RNA integrity number was over 7.0. Double-stranded cDNA libraries (RNA-sequencing libraries) were prepared using a SMART-seq HT kit (Clontech) using a DNBSEQ MGIEasy Universal Library Conversion kit (MGI Tech) according to the manufacturer’s instructions. RNA-sequencing libraries were sequenced using paired-end reads (150 nt of read1 and read2) on a DNBSEQ-G400RS instrument (MGI Tech). Obtained raw reads were trimmed and quality-filtered using Trim Galore! (version 0.4.4; Babraham Institute), Trimmomatic (version 0.36; Bolger et al., 2014), and cutadapt (version 1.16) software (Martin, 2011). Trimmed reads were then mapped to the mouse GRCm38 genome using STAR (version 2.7.2b; Dobin et al., 2013). Reads on annotated genes were counted using featureCounts (version 1.6.1; Liao et al., 2014). Fragments per kilobase of exon per million reads mapped (FPKM) values were calculated from mapped reads by normalizing total counts and transcripts. Differentially expressed genes were detected using the DESeq2 package (version 1.20.0; Love et al., 2014). The list of differentially expressed genes detected by DESeq2 (basemean >5 and fold-change <0.25, or basemean >5 and fold-change >4) was used for GO enrichment analysis by the clusterProfiler package (Yu et al., 2012).

### GSEA

GSEA was performed using RNA-sequencing data (FPKM+1) with GSEA_4.1.0 software (Broad Institute). Gene sets were obtained from the Molecular Signatures Database (MSigDB) v4.1.0 distributed at the GSEA website (https://www.gsea-msigdb.org/gsea/msigdb/) or previous reports (Brown et al., 2006).

### Statistical analysis

Statistical analyses were performed using Microsoft Excel or Prism software version 3 (GraphPad). A two-tailed Student’s *t* test was used for statistical analyses of two-group comparisons. Multigroup comparisons were performed using one-way ANOVA followed by the Tukey–Kramer multiple comparisons test. The criterion of significance was set at P < 0.05. Results with biological replicates were expressed as means ± SD, and data with technical replicates were expressed as means ± SEM. Blinding or randomization of the groups was not performed and no data were excluded. No statistical methods were used to estimate sample size.

### Online supplemental material

Fig. S1 shows differences between M-B cells and other CD11b^+^ B cell subsets. Fig. S2 shows experimental strategies for intra-BM transplantation of B cell–lineage cells and the unnecessity of TLR4 expressed by B cell precursors in the development of M-B cells. Fig. S3 shows the numerical changes of Tregs and plasma cells in the BM after LPS treatment. Fig. S4 shows the impact of B cell–derived IL-10 on levels of serum cytokines and apoptosis of BM myeloid cells after LPS treatment and G-CSF–induced myelopoiesis. Fig. S5 shows the effect of IL-10 in myeloid cell differentiation.

## Data Availability

The RNA-seq data have been deposited in the National Center for Biotechnology Information Gene Expression Omnibus under accession no. GSE222966.
